# Devising focused strategies to improve organ donor registrations: A cross-sectional study among professional drivers in coastal South India

**DOI:** 10.1371/journal.pone.0209686

**Published:** 2018-12-21

**Authors:** Akshay Thimmappaiah Jagadeesh, Anushree Puttur, Soumayan Mondal, Sufyan Ibrahim, Anurupa Udupi, Lokadolalu Chandracharya Prasanna, Asha Kamath

**Affiliations:** 1 Undergraduate Students, Kasturba Medical College, Manipal Academy of Higher Education, Manipal, Karnataka, India; 2 Department of Anatomy, Kasturba Medical College, Manipal Academy of Higher Education, Manipal, Karnataka, India; 3 Department of Statistics, Prasanna School of Public Health, Manipal Academy of Higher Education, Manipal, Karnataka, India; University of Toronto, Rotman School, CANADA

## Abstract

**Background:**

In India, annually, 500,000 people die due to non-availability of organs. Given the large proportion of brain death amongst road accident victims, any improvement in organ donation practices amongst this cohort could potentially address this deficit. In this study, we identify the potential areas for intervention to improve organ donation amongst professional drivers, a population more likely to suffer from road accidents.

**Methods:**

300 participants were surveyed using a structured, orally-administered questionnaire to assess knowledge, attitudes and practices regarding organ donation. Multivariate analysis was performed to identify key variables affecting intent to practice.

**Results:**

Nearly half our participants had unsatisfactory knowledge and attitude scores. Knowledge and attitude was positively correlated, r_s_ (298) = .247, *p* < .001, with better scores associated with a higher likelihood of intent to practice organ donation [AOR: 2.23 (1.26–3.94), *p* = .006; AOR: 12.164 (6.85–21.59), *p* < .001 respectively]. Lack of family support and fear of donated organs going into medical research were the key barriers for the same [AOR: 0.43 (0.19–0.97), *p* = .04; AOR: 0.27 (0.09–0.85), *p* = .02 respectively].

**Conclusion:**

Targeted health-education, behaviour change communication, and legal interventions, in conjunction, are key to improving organ donor registrations.

## Introduction

It has been long established that, for patients with end-stage organ-specific disease, transplantation offers the most functional status if not the only definitive treatment possible.[1] However still, in India, every year nearly 500,000 people die because of non-availability of organs,[2] largely attributed to a limited number of organ donors in the country.[3] Surprisingly, for a country with a population of over 1.32 billion, in 2016, the statistics showed a deceased donor transplantation rate of 0.34 per million population.[4] This statistic is very low compared to Europe’s 21.53 deceased organ donors per million population.[5] This could be attributed to the lack of knowledge and understanding about organ donation, religious attitudes, and superstitious beliefs that have generated fear and mistrust among the masses. [6,7]

A 2008 study has shown that more than 100,000 people die due to road traffic accidents (RTA) in India every year, with a significant portion of deaths occurring due to traumatic brain injury.[8] A prior study done in 2005–09, in the Udupi district of Karnataka, showed that brain injury accounted for 75% of all deaths due to RTAs (*N* = 344).[9] A brain death allows for a higher likelihood of viable organs in the deceased. Given their occupational exposure to stresses and fatigue, professional taxi drivers are at an increased risk of RTAs, constituting 7–27% of all RTAs (*N* ranges from 6,259–480,652) with about 5–12% of them proving fatal, albeit there exist geographic and temporal variations.[10–13] As drivers are more likely to be involved in an RTA a large proportion of the organ deficit could be addressed by drivers who are educated and aware of the need for organ donation. Additionally, of the majority of the knowledge-attitude-practice (KAP) studies in relation to organ donation done in India, most have been done on either dental and medical students,[14–16] or other cohorts with education qualifications beyond secondary school.[17] A handful of studies have also been done on patients and their relatives.[3,18,19] However, studies focusing on groups that have a higher likelihood of addressing the current organ donor deficit have yet to be explored.

The present study assesses the knowledge, attitude and beliefs towards organ donation, and the factors affecting willingness to donate organs among professional drivers residing in coastal South India. The results suggest that, by improving knowledge, and eliminating certain negative attitudes, an atmosphere conducive for organ donation could be created among the driver population. This could enhance the rates of organ donor registrations and donations per year. This warrants further interventional studies to confirm the same.

## Methods

### 2.1 Study population

This was a cross sectional study, carried out in the Udupi district of Karnataka, India from July, 2016 to February, 2017. A total of 300 male professional taxi (three or four-wheeler) drivers were recruited via convenient sampling from all the taxi stands in the district in order to make the sample more representative. They were surveyed using a questionnaire to determine their knowledge, attitude and practices regarding organ donation. The sample size was calculated anticipating 59.6% of the professional drivers to have a willingness to donate with a relative precision of 10% at 95% confidence level and accounting for a 15% non-response rate.[3] All professional drivers, except those already holding an organ donor card, were included in the study.

### 2.2 Data collection and management

The primary outcome of the study was to assess the intent to donate ones’ organs. All variables thought to affect the outcome were collected via personal interviews using a structured non-disguised questionnaire. The questionnaire was constructed after due consideration of published literature.[3,17,18,20] It was finalized after a pilot study, to ensure comprehensibility among study subjects, and assess for reproducibility and validity. Personal data collected included age, educational status, religion, and place of residence (rural or urban). Six items on the questionnaire were used to assess the participants’ knowledge regarding organ donation including the possibility of living or deceased donations of different organs, and relevant legislation. Fifteen items were used to assess the participants’ attitude towards organ donation: divided into two sections assessing their preference of donation and apprehensions that may prevent them from signing up for an organ donor card. Two items were used to assess practice parameters: one identified participants who practiced organ donation in the past; another, assessed their intent to practice, as determined by their willingness to sign up for an organ donor card. This question was asked after explaining to them that signing-up for an organ donor card would entail self-donation of organs after death. Interviews were conducted by investigators fluent in the local language (Kannada), with care taken to ensure that no information regarding organ donation was presented to the participants during the interview.

### 2.3 Statistical analysis

All data was entered and analyzed using the Statistical Package for Social Services (SPSS) version 15. The complete dataset can be found in [21]. Twenty-one items on the questionnaire were used to define the levels of knowledge and attitude pertaining to organ donation. One point was given for every correct, affirmative, or positive response—detailed information on the scoring system is given in S1 Table. The maximum scores calculated, were 20 and 15, for knowledge and attitude scores respectively. The outcome variable, response to willingness to sign-up for an organ donor card, was recorded dichotomously as positive or negative—including no, not right now, and not applicable (for all participants unaware of deceased donor organ donation).

Descriptive analyses were performed to characterise the data. Chi-Square or Fisher Exact tests, depending on sample size, were done for associations between categorical variables. Correlations between continuous variables were assessed using Spearman’s rank-order correlation. A binary logistic regression model was constructed to identify the independent predictors of intent to practice organ donation. All statistical analyses were two-tailed, set at a confidence interval of 95% with a *p* value of < .05 considered statistically significant.

### 2.4 Ethical considerations

The study protocol was approved by the Institutional Ethics Committee of the Kasturba Hospital and Kasturba Medical College, Manipal Academy of Higher Education (IEC No: 474/2016) and was conducted according to its guidelines. All study participants were informed of the voluntary nature of the study and gave a written informed consent.

## Results

The study included a total of 300 male participants, all drivers by profession, with a mean age of 38.28 ± 11.18 years. 276 (92%) participants had an educational qualification of high school (10^th^ class) or above. A majority of the participants, 274 (91.3%) were Hindus.

### 3.1 Questionnaire parameters regarding organ donation

The survey questions, and the absolute and relative (%) number of correct, affirmative, or positive responses on knowledge, attitude, and practices regarding organ donation are given in S2 Table.

#### 3.1.1 Knowledge

**Fig 1:**
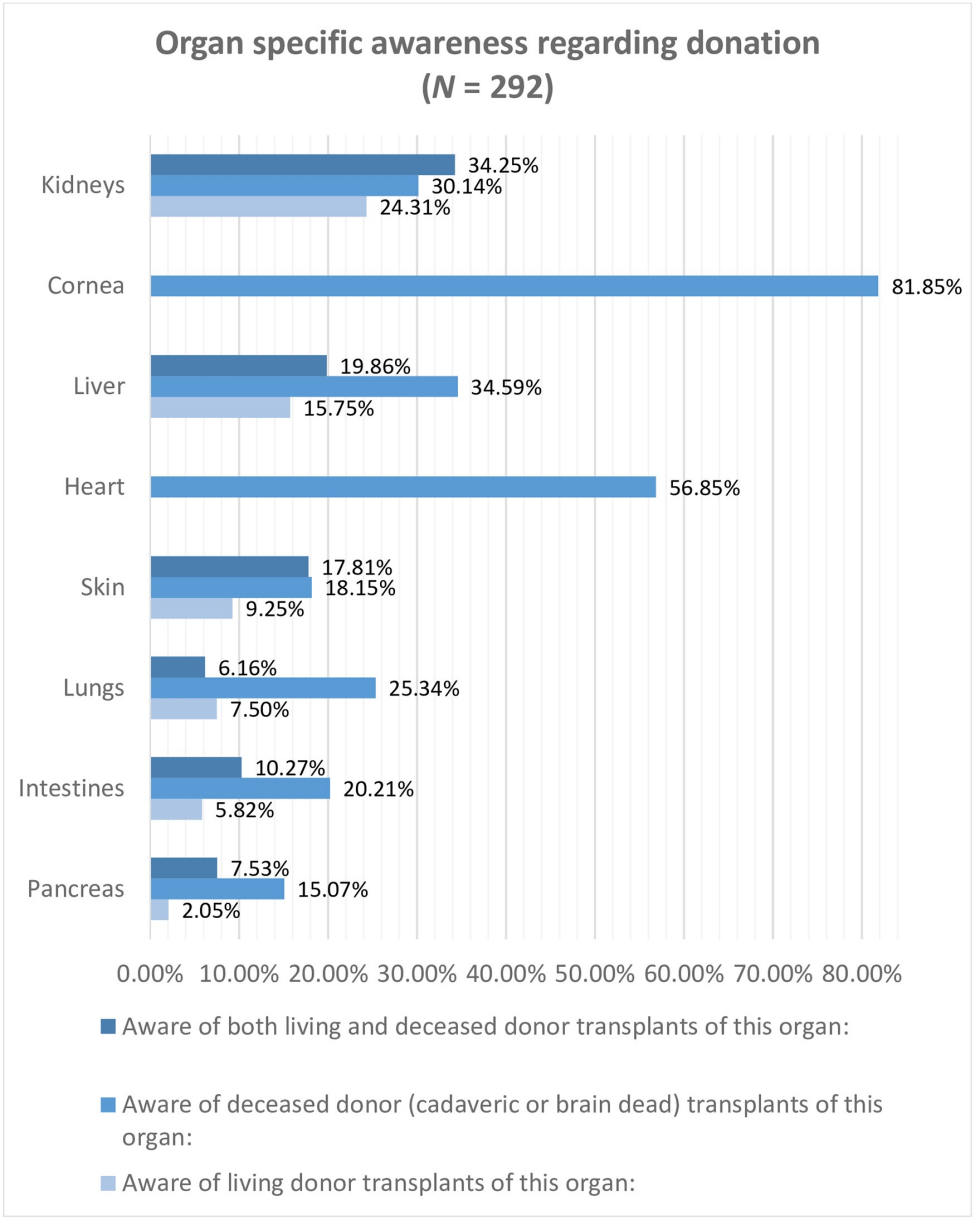
Shows percentage of study population aware of the possibility of donor transplants for different possible organs.

Nearly all the study participants, 292 (97.33%) had heard about organ donation. Less than half these participants, 133 (45.55%), were correctly aware that some organs could be donated when alive and some others upon death (cadaveric or brain-dead donation). A sizeable proportion, 244 (83.56%) were aware of the possibility of a deceased donor transplant. However, few participants, 31 (10.62%) responded that organs can be donated only when alive. Organ specific awareness regarding donation is shown in Fig 1. Awareness regarding kidney donations were higher than any other organ. A majority of the population (*N* = 292), 88.70% were aware about either living or deceased kidney donor transplants. Awareness regarding corneal donor transplants was also high at about 81.85%. A good majority, 70.20% were aware about either living or deceased liver donor transplants. Though, partial lung, pancreas, or intestine, and skin, living-donor transplants are now medically possible, these are not permitted in India as per the Transplantation of Human Organs (Amendment) Act 2011,[22] this distinction between possibility and legality of living donations of these organs was not made. Among those aware of living donor transplants, 124 (75.61%) were unaware that living donation entails medical risks to the donor. Nearly all these individuals, 160 (97.56%), were aware that living altruistic (directed or non-directed) donations are legal in India. However, more than a fourth of those aware of organ donation, 81 (27.74%), were unaware that accepting monetary or other benefits for donation of organs is illegal in India.

#### 3.1.2 Attitude

Nearly everyone, 275 (94.18%) said that they would support their kin’s decision to become an organ donor. A majority, 219 (75.00%) said that they would consent to a non-directed altruistic donation of a deceased relatives’ organs. Among the 211 (72.26%) participants who reported that they would like to donate their organs, a mere 11 (5.21%) said that they would donate their organs only to family or close friends; however, 50 (23.70%) had certain age restrictions concerning who would receive their organs; 70 (33.18%) expressed a preference to donate only to mentally sound recipients; a diminutive fraction, 2 (1%) had religious restrictions concerning the recipients. Over half of those willing to donate, 114 (54.03%) responded that they preferred deceased donation, 25 (11.85%) preferred living donation, while 72 (34.12%) had no preferences. A majority of participants, 207 (70.89%) agreed that there is a lack of awareness regarding organ donation among the general masses in the country.

**Fig 2:**
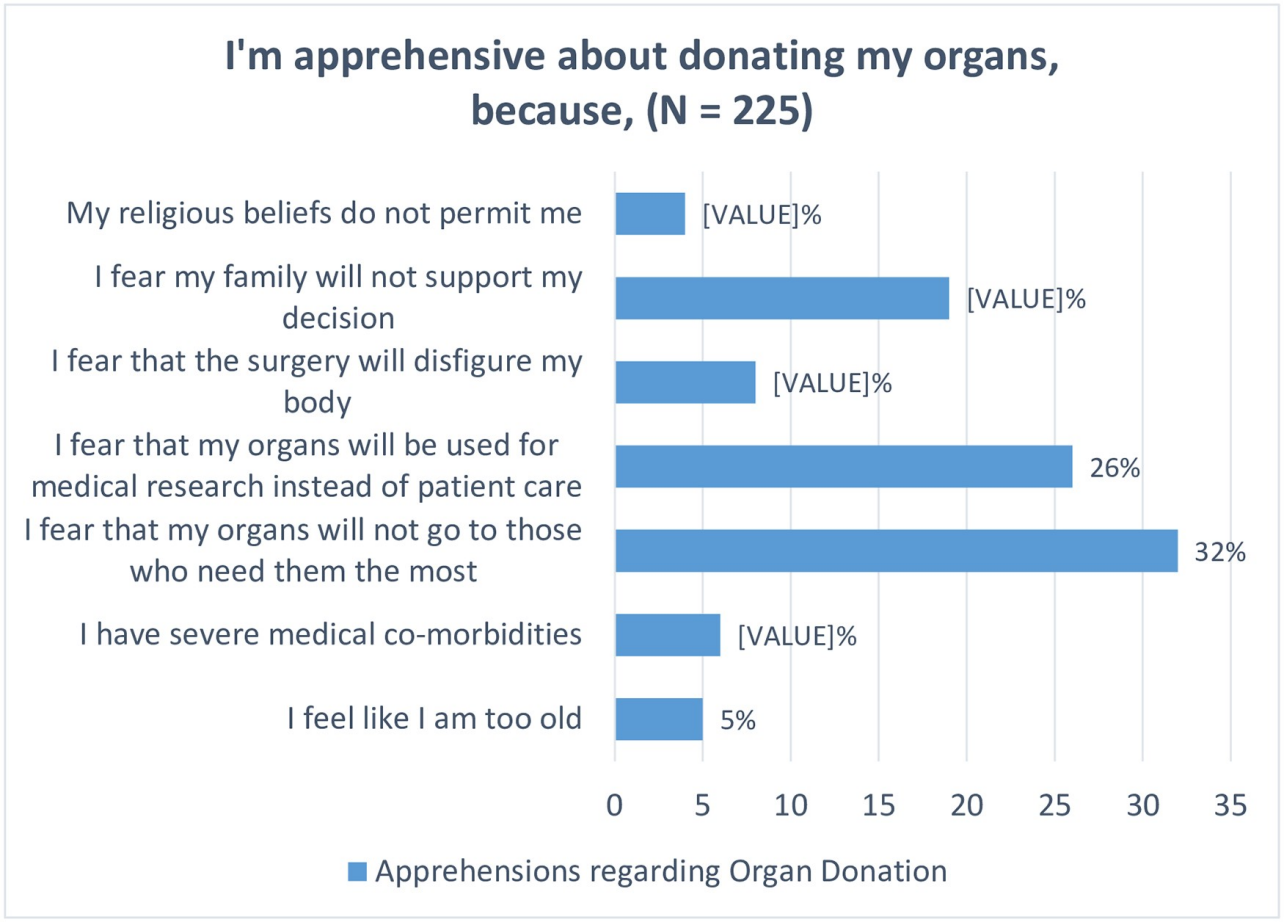
Bar-graph representing the apprehensions regarding organ donation.

**3.1.2.1 Barriers to organ donation and their associations with subject socio-demographic characteristics**. Of the 292 people who were aware of the concept of organ donation, 133 (45.86%) had at least one apprehension regarding organ donation, shown in Fig 2. Lack of family support, and distrust towards the health-care system, as indicated by fear that donated organs would be used for medical research and/or would not reach those who need it most, constituted the largest bulk, 76.89% of all barriers to organ donation (*N* = 225).

Chi-square tests (or Fishers Exact tests depending on sample size) were performed to determine associations, if any, between subject demographic characteristics and apprehensions regarding organ donation. Within this cohort of professional drivers, the participants’ age, educational qualification, and place of residence did not bear any significant statistical association with having any apprehensions regarding organ donation (Refer S1 File).

The participants’ religion, however, had significant associations with certain apprehensions: (a) 37.50% of Muslims (*N* = 16) responded that their religious beliefs do not permit them to donate their organs, compared to the much lesser 1.45% of participants of other faiths (*N* = 276), giving an odds ratio (OR) of 40.80 (9.92–167.75), *p* < .001. Among Muslims, those who perceived organ donation to be permissible by their religion had a higher knowledge score than those who didn’t, (11.10± 4.77) as opposed to (7.17 ± 1.72) with a mean difference of 3.933. However, this difference was not significant, t (14) = 1.92, *p* = .08. To evaluate the ability of our study to detect a significant difference in this regard, we performed a post-hoc power analysis with the calculated effect size, Cohen’s *d* = 1.096. The power so calculated (51%) warrants the need for further studies with a larger Muslim population to confirm the same.

(b) 43.75% of Muslims responded that their family might not support their decision to donate organs, larger than the 12.68% of participants of other faiths, giving an OR of 5.36 (1.87–15.30), *p* = .003.

#### 3.1.3 Practice

Only 3 (1.00%) participants had donated organs in the past. Half the study population, 151 (50.33%), showed an intent to practice organ donation, responding that they are willing to sign-up for an organ donor card.

### 3.2 Knowledge and attitude go together

#### 3.2.1 Scoring on questionnaire parameters

The participants’ (*N* = 300) median score on knowledge parameters was 9 (interquartile range (IQR): 7–12), with only 1 participant having a perfect score (calculated out of a possible 20); we used a cut-off score of ≥9 to describe a ‘*satisfactory’* score on knowledge. The median score on attitude parameters was 12 (IQR: 8–14), with 12.67% of participants (*N* = 300) having a perfect score (calculated out of a possible 15); we used a cut-off score of ≥12 to describe a *‘satisfactory’* score on attitude. This way 182 (60.67%) and 172 (57.33%) of participants had satisfactory scores on knowledge and attitude parameters, respectively.

#### 3.2.2 Correlation

A Spearman’s rank-order correlation was run to assess the relationship between scores on knowledge and attitude parameters regarding organ donation among the participants (N = 300). An increase in the participants’ scores on knowledge parameters was significantly associated with that of attitude parameters, indicating a strong positive correlation, r_s_ (298) = .247, *p* < .001.

### 3.3 Associations between demographic characteristics and *satisfactory* knowledge, attitude, and practice parameters

The demographic characteristics of the population and their association with the participants’ scores on knowledge and attitude parameters, and their intent to practice organ donation is given in Table 1. Bivariate analysis revealed no significant statistical association between the participants’ age, religion, or place of residence with a satisfactory score on either knowledge or attitude parameters, or their willingness to sign-up for an organ donor card. Educational qualification, however, had an association with *satisfactory* scores on knowledge, but not attitude or practice parameters. Participants with an education up to pre-university education (11^th^ or 12^th^ class) or higher were more likely to secure *satisfactory* knowledge scores than those who studied up to 10^th^ Class or less, with an OR of 2.45 (1.43–4.19), a significant association, χ^2^ (1) = 10.93, *p* < .001. On multivariable analyses with age, religion, and locality as covariates, this association remained significant giving an adjusted odds ratio (AOR) of 2.55 (1.44–4.50), *p* = .001; further, participants from an urban locality were more likely to get a *satisfactory* score on knowledge parameters with an AOR of 1.72 (1.05–2.82), *p* = .03. No other demographic characteristic was significantly associated with *satisfactory* knowledge or attitude scores, or willingness to sign-up for an organ donor card.

**Table 1:** Study population demographics and their association with the participants’ scores on knowledge and attitude parameters, and willingness to sign-up for an organ donor card.

Demographic characteristics	Total responses	Satisfactory parameter scores on				Are you willing to sign up for an organ donor card?	
		Knowledge		Attitude		Positive responses	
	*N* = 300	162 (54.00%)	*p value*	172 (57.33%)	*p value*	151 (50.33%)	*p value*
*1*. *Age*							
19–29	75 (25.00%)	51 (17.00%)	.50	45 (15.00)	.77	38 (12.67%)	.43
30–39	95 (31.67%)	54 (18.00%)		58 (19.33%)		50 (16.67%)	
40–49	77 (25.67%)	43 (14.33%)		40 (13.33%)		33 (11.00%)	
50–59	40 (13.33%)	26 (8.67%)		22 (7.33%)		21 (7.00%)	
60–73	13 (4.33%)	8 (2.66%)		7 (2.33%)		9 (3.00%)	
*2*. *Educational status*							
Illiterate & Primary school	24 (8.00%)	9 (3.00%)	.002*	10 (3.33%)	.25	10 (3.33%)	.26
Middle & High school (up to 10^th^ Class)	182 (60.67%)	103 (34.33%)		111 (37.00%)		100 (33.33%)	
PUC (11^th^ or 12^th^ Class)	65 (21.67%)	50 (16.67%)		34 (11.33%)		28 (9.33%)	
Graduate degree / higher	29 (9.67%)	20 (6.67%)		17 (5.67%)		13 (4.33%)	
3. *Religion / faith*							
Hinduism	274 (91.33%)	165 (55.00%)	.47^f^	158 (52.67%)	.86^f^	136 (45.33%)	.86^f^
Christianity	10 (3.33%)	8 (2.67%)		5 (1.67%)		6 (2.00%)	
Islam	16 (5.33%)	9 (3.00%)		9 (3.00%)		9 (3.00%)	
4. *Place of residence*							
Rural	159 (53.00%)	103 (34.33%)	.12	88 (29.33%)	.46	82 (27.33%)	.50
Urban	141 (47.00%)	79 (26.33%)		84 (28.00%)		69 (23.00%)	

PUC: Pre-university College; Data is expressed as frequencies and percentages; *p* values are calculated using the Chi-square test of independence (unless specified);

^f^Fisher’s exact test;

*Statistically significant finding

### 3.4 Satisfactory scores are associated with an intent to practice to organ donation

On bivariate analysis, of the total 151 participants willing to sign-up for an organ donor-card, those with *satisfactory* knowledge scores, 110 (70.85%), were more likely to sign-up for organ donor cards as opposed to those without, with an OR of 2.87 (1.83–4.64), yielding a significant association, χ^2^ (1) = 18.91, *p* < .001. Participants with satisfactory attitude scores, 128 (84.77%), were also more likely to sign-up for organ donor cards, with an OR of 13.28 (7.53–23.40), also a significant association, χ^2^ (1) = 93.55, *p* < .001.

On multivariate analysis, the associations of *satisfactory* knowledge and attitude scores with the intent to practice organ donation remained significant [AOR: 2.23 (1.26–3.94), *p* = .006; AOR: 12.164 (6.85–21.59), *p* < .001 respectively).

### 3.5 Specific parameters that affect one’s intent to practice organ donation

A binary logistic regression was constructed to ascertain the association between the participants’ knowledge and attitude parameters, and the likelihood of them signing-up for an organ donor card (Table 2). Only those knowledge and attitude parameters thought to be relevant to deceased non-directed altruistic organ donation, with responses from participants aware of the concept of organ donation, 292 (97.33%) of the study population, were considered for the analyses. An initial univariate analysis was performed to identify the covariates of interest (taking the conventional *p* < .25 based on the Wald Chi-squared test statistic). We then assessed if the remaining variables (*p* > .25 on univariate analysis) could have a relevant effect on the model by including them one at a time, assessing each model for both the assumption of multicollinearity and best fit. A parameter corresponding to family support affecting ones’ decision to become an organ donor, not significant in the univariate analysis, was included in the final model. Though not statistically significant, its’ theoretical relevance deemed its’ inclusion. A likelihood ratio test performed to compare the fit of the models with and without the said variable, further justified its inclusion, χ^2^ (1) = 4.11, *p* = .04. When all 292 responses were analysed, there were 6 outliers with studentized residuals ≥ 2.5 SD, excluded from further analysis. A thorough analysis of these six participants’ responses, supporting its exclusion is given in S3 Table.

**Table 2:** Predictors of willingness to sign up for an organ-donor card using univariate and ultivariate analyses.

Knowledge / Attitude parameters	Are you willing to sign up for an organ donor card?			Uni-variate	Multivariate analysis	
	Total *N* = 292	Positive response	Negative responses	*P* value*	OR (CI)	*p* value*
*1*. *Aware of the possibility to donate one’s organs after death*? *i*.*e*. *aware of deceased donor (cadaveric or brain dead) transplants*?						
Yes	244 (83.56%)	145 (49.66%)	95 (33.90%)	< .001	18.71 (5.50–63.67)	< .001^ϕ^
No	48 (16.44%)	6 (2.05%)	42 (14.39%)			
*2*. *Aware that*, *with respect to organ donation in India*, *it is illegal for donors or their families to accept*, *monetary or other benefits from the recipient*?						
Yes	211 (72.26%)	117 (40.07%)	94 (32.19%)	.04	1.97 (0.92–4.22)	.08
No	81 (27.74%)	34 (11.64%)	47 (16.10%)			
*3*. *If asked to donate organs from a deceased close family member*, *would you agree*?						
Yes	219 (75.00%)	127 (43.49%)	92 (31.51%)	< .001	1.39 (0.58–3.34)	.46
No / Don’t know	73 (25.00%)	24 (8.22%)	49 (16.78%)			
*4*. *If you were to donate your organs*, *what sort of donation would you prefer*?						
Living donation	25 (8.56%)	10 (3.42%)	15 (5.14%)	< .001	1.64^a^ (0.51–5.22)	.41
Deceased donation	114 (39.04%)	79 (27.05%)	35 (11.99%)			
Either is fine	72 (24.66%)	56 (19.18%)	16 (5.48%)			
NA—I do not wish to donate my organs	81 (27.74%)	6 (2.05%)	75 (25.69%)			
*5*. *I feel my family may not support my decision to donate my organs*:						
Yes	42 (14.38%)	23 (7.88%)	19 (6.50%)	.67	0.43 (0.19–0.97)	.04^ϕ^
No / Don’t know	250 (85.62%)	128 (43.84%)	122 (41.78%)			
*6*. *I have concerns that my organs be used for medical research rather than for patients*:						
Yes	58 (19.86%)	22 (7.53%)	36 (12.33%)	.02	0.27 (0.09–0.85)	.02^ϕ^
No / Don’t know	234 (80.14%)	129 (44.18%)	105 (35.96%)			
*7*. *I have concerns that my organs will not go to those patients who need it most*:						
Yes	73 (25.00%)	30 (10.27%)	43 (14.73%)	.04	1.49 (0.49–4.57)	.48
No / Don’t know	219 (75.00%)	121 (41.44%)	98 (33.56%)			

Data is expressed as frequencies and percentages; Negative responses include no, not right now, and not applicable; OR: Odds Ratio; CI: Confidence Intervals; NA: Not Applicable;

^a^deceased vs living donations;

*Wald test;

^**ϕ**^Statistically significant in the regression model

The final regression model constructed was statistically significant, χ^2^ (9) = 187.23, *p* < .001, correctly predicting responses in 83.57% of the cases, and accounting for 64.06% (Nagelkerke *R*^2^) of the variance. Hosmer and Lemeshow test indicated a good fitting model, χ^2^ (7) = 1.76, *p* = .97. Of the seven predictor variables, three were statistically significant. Participants who were aware of the possibility of deceased donor transplants had 18.71 times the odds for signing-up in contrast to those who lacked this awareness, *p* < .001. Participants who were uncertain about their family’s support of them donating their organs had 57% lower odds for signing-up compared to those who were confident of their family’s support, *p* = .04. Among those who expressed concerns about their organs going into research rather than patient-care had 73% lower odds for signing-up compared to those without such concerns, *p* = .02. Other variables, though not statistically significant, suggest that participants with favourable responses on knowledge and attitude parameters i.e. correct, affirmative, or positive responses, have higher odds of signing up for organ donor cards.

## Discussion

With the goal of identifying specific problem areas for more focused interventional approaches to improve rates of organ donor card registrations, the present study explored the knowledge deficit, and delineated the attitude and associated practices pertaining to organ donation.

### 4.1 Knowledge parameters pertaining to organ donation

97.33% of our study population (*N* = 292) had heard about the term ‘organ donation’, comparable to the 100% reported by a study among patients in coastal South India,[3] higher than the 74.90% and 86% reported from studies done on general populations in northern and western India respectively.[19,23] This could perhaps be attributed to Udupi district’s high literacy rate (86.20%),[24] and the educational status of our study population, >92% had an education of 10^th^ class or higher. (Refer 3.1.1) Regions with higher literacy rates usually have higher awareness regarding the term ‘organ donation’, as reported by studies done on general populations in South India.[3,20]

Awareness regarding the possibility of deceased donor transplants was higher than that for living donor transplants, 244 (83.56%), as opposed to 164 (56.14%). Participants who were aware of deceased donor transplants had 18.71 times the odds for signing up, in contrast to those who lacked this awareness, *p* < .001. (Refer Table 2) A high awareness is perhaps, both a cause and consequence of the increased deceased donor transplantations in the state, from a rate of 0.28 (per million population) in 2012 to 0.60 in 2014, as reported by Mohan Foundation (an NGO based in India).[25] Only a fourth of those aware of living donor transplants, 40 (24.39%), were aware that it entails medical risks to the donor, lower than the 40% reported by another study in a nearby region.[3] Further, awareness regarding the possibility of kidney and corneal donations were higher than other organs, >80% (*N* = 292); followed by awareness regarding liver and heart donations, at 70.20% and 56.85%, respectively. (Refer Fig 1) This is in agreement with other studies conducted in South India.[3,20] Awareness pertaining to organs could be attributed to media coverage, efforts by government and NGOs,[3,20] and the fact that health-centers that handle transplantations of other organs are scarce in the country.

#### 4.1.1 Knowledge regarding the laws surrounding organ donation in India

Almost all aware of living donations, 160 (97.56%), knew that living altruistic (directed or non-directed) donations are legal in India. Further, a majority of the population, 211 (72.26%), were aware that accepting monetary or other benefits for organ donation is illegal as per Indian law. These figures are higher than the 75.20%, and 58.10%, respectively, reported from a study in coastal South India.[3] In principle, the illegality of monetary or other incentives to the donor, is to ensure that donations stem purely out of altruistic motives rather than commercial ones, in a view to combat organ trafficking.[26] Participants who were correctly aware that it is illegal to accept incentives for donations, were significantly more likely to sign-up for an organ donor card, OR: 1.72 (1.03–2.89), *p* = .04. We speculate whether this finding indicates that those with an intent to practice organ donation, do so purely out of altruism and solidarity.[27] However, this association was not significant on multivariable analysis, AOR: 1.97 (0.92–4.22), *p* = .08. (Refer Table 2) In actuality, Indian laws have failed to check commercial donations that the poor strata of the society, in collusion with the people who harvest organs, try to pass off as altruistic ones.[26]

#### 4.1.2 Correlating socio-demographic parameters with knowledge

**4.1.2.1 Religion—does more awareness make organ donation permissible?**: In our study, 37.50% of Muslims (*N* = 16) responded that their religious beliefs do not permit them to donate their organs, compared to the 1.45% of participants of other faiths (*N* = 276), giving a statistically significant difference, *p* < .001. This correlates with the finding that in 2013–2016, a nearby state in Southern India, reported no Muslim donors among the >1000 organs transplanted.[28] These results are in agreement with donation rates in Islamic countries, where in addition to logistical difficulties, religious beliefs constitute a large barrier.[29] A recent study among Muslims in Palestine found that, the belief that organ donation was permissible in Islam was significantly associated with a high level of knowledge regarding organ donation.[30] In our study, we found Muslims who perceived organ donation to be permissible by their religion to have a higher knowledge score than those who didn’t, (11.10± 4.77) as opposed to (7.17 ± 1.72) with a mean difference of 3.933, *p* = .08. The near significant value may be attributed to the relatively small proportion (5.33%) of Muslims in our study population (*N* = 300). (Refer 3.1.2.1) Though several Islamic countries have passed religious rulings endorsing organ donation, there are wide discrepancies within different sects of the community, compelling Muslims to seek the local Imams’ advice when faced with question of donating or receiving organs.[31] This places these local Islamic scholars at a key position in influencing organ donor rates in these communities, making them effective targets for health education.

**4.1.2.2 A higher educational status is indirectly associated with the intent to practice organ donation**: A recent study among the general population in a nearby city, found those with higher educational status, and urban background to be significantly more likely to respond positively to organ donor card registrations.[18] Though we did not find such associations, we found participants with a pre-university education or higher, and those from an urban background to be more likely to secure *satisfactory* knowledge scores, [AOR: 2.55 (1.44–4.50), *p* = .001; AOR: 1.72 (1.05–2.82), *p* = .03 respectively]. Further, those with *satisfactory* knowledge scores, 110 (70.85%), were more likely to sign up for organ donor cards, AOR: 2.23 (1.26–3.94), *p* = .006, results that were comparable to the study done in Bangalore.[18] Our findings suggest an indirect relationship between educational status and intent to practice organ donation. They also indicate that improving knowledge regarding organ donation may prove beneficial in improving donor registrations. Further, health education in organ donation when introduced among school children, in addition to the obvious increase in knowledge, is said to facilitate open discussions amongst family members which is a step forward in acquiring family support, as reported by a 2017 study in the Netherlands.[32]

### 4.2 Importance of family—Patient—Doctor dynamics in organ donation

A study in the USA revealed patients who were more likely to discuss their decision to donate with their families, had lower levels of distrust towards health-care professionals.[33]

#### 4.2.1 Family support—A psychological necessity for the patient and a legal requirement by the state

We found satisfactory attitude scores to be significantly associated with the intent to practice organ donation AOR: 12.164 (6.85–21.59), *p* < .001. Apprehensions regarding lack of family support was found to be one of the main factors negatively affecting attitudes scores. Further, those with this apprehension had a 57% lesser likelihood of signing up for an organ donor card when compared to those without, *p* = .04 (Refer 3.1.2.1). This apprehension was more prevalent in Muslim communities, 43.75% vs the 12.68%, *p* = .003. Studies have shown that the greatest factor contributing to low levels of deceased donation is non-consent by the family. A study done in Wales, where, as in India, consent by the next-of-kin is mandatory for deceased organ donations regardless of ones’ organ donor card status,[34] showed that 10% of registered deceased donors were overruled by their families.[35] A study conducted in 16 U.S. tissue banks, estimates that 30% of the families of tissue donor-eligible patients, refuse donation.[36] The same study concluded that families with favourable knowledge and attitude parameters regarding donation had a 10 times greater likelihood of consenting to donate their deceased next of kin’s tissue. As comparable with our study population, a study in South India reported 83% of subjects believe that their family has the right to determine whether they will be donating their organs or not. [17] These studies highlight the importance of donor-family dynamics on the aspect of organ donation.

#### 4.2.2 Distrust towards the health-care system: The largest barrier to organ donation

Our results also highlight an increasingly common theme of distrust among the public towards the health-care system. A 2017 study in Mexico, found, *distrust in the transplant process* to be one of the key hurdles that prevent people from donating their organs after death. [37] In our study, distrust towards the health-care system constituted 130 (57.78%) of all apprehensions (Refer Fig 2). Nearly a quarter of all participants unwilling to sign up were apprehensive because they believed their organs would be used for medical research rather than for patient-care. Those with this apprehension had a 73% lesser likelihood of signing up for a donor card compared to those without, *p* = .02 (Refer 3.2.2.1). Additionally, 30% of all unwilling to sign up were concerned about their organs not going to those who need them most (Table 2). This distrust could potentially stem from numerous factors including beliefs that doctors are more concerned about their own financial well-being than the patients’ health, and negative media coverage regarding doctors and their practice. [38] Physician distrust produces poorer health outcomes as it leads to an increased reluctance in the community to accept a physician’s word as truth. A recent study conducted in the USA, found that many patients believed recipients of kidney-transplants were often wealthier, and had a higher educational and social status.[39] This distrust in the transplant process equity is considered a major cause for the disparity between supply and demand for kidney transplantation. The same study proposed increased transparency in doctor-patient relationships, and more time devoted to educating the patient on, the process of organ donation as well as organ transplantation, as an approach reduce this distrust. [39]

These results highlight the need for better donor-family and donor-physician relationships: two interdependent aspects that need to be thoroughly considered in the planning of interventional strategies to improve organ donor rates.

### 4.3 Intent to practice organ donation

Half the study population, 151 (50.33%), showed an intent to practice organ donation, responding that they are willing to sign-up for an organ donor card, marginally lower than the 59.60% reported by a study conducted on general population in coastal South India.[3]

#### 4.3.1 Improving organ donor card registrations: What can be done?

**Fig 3:**
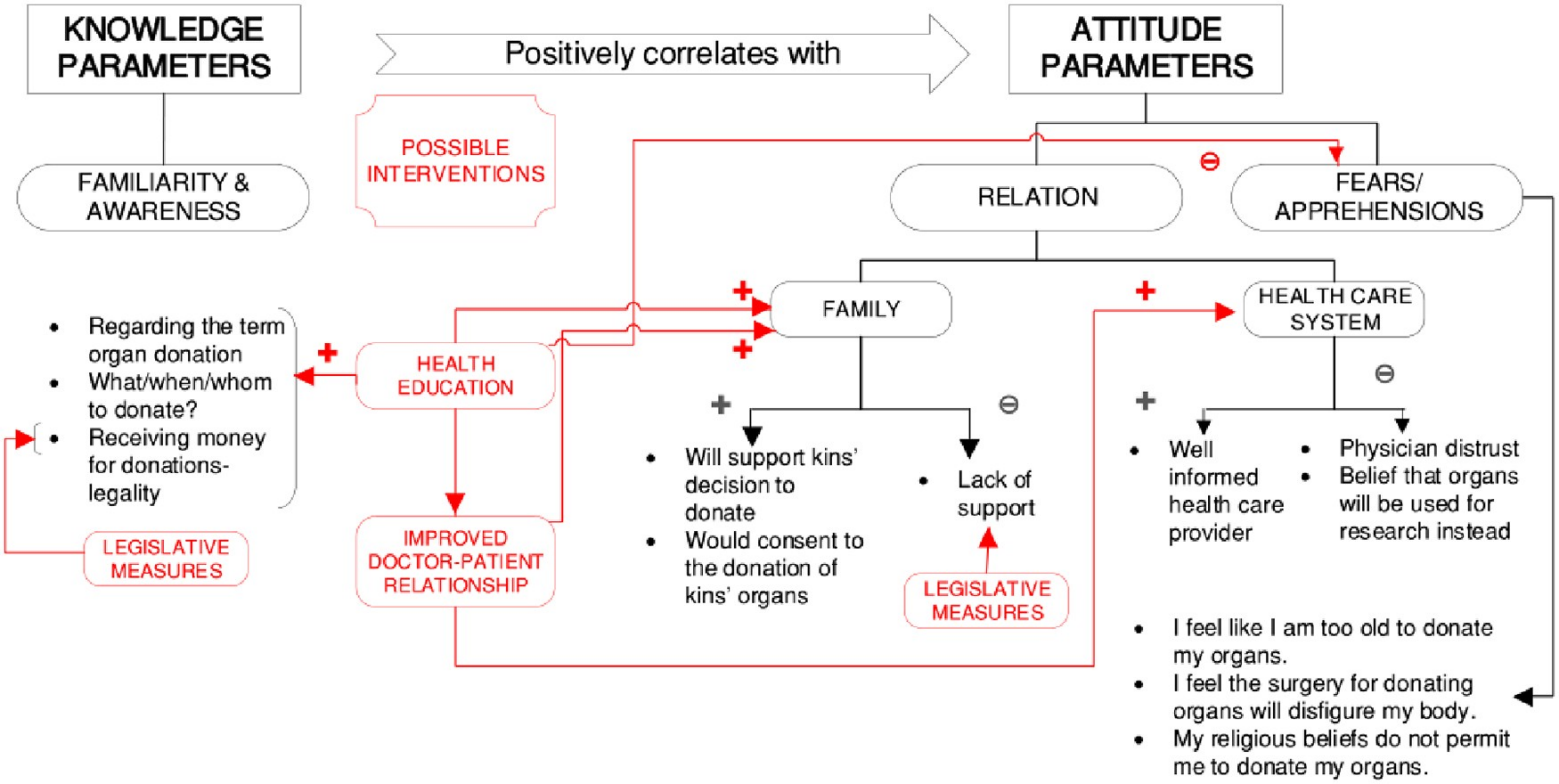
Willingness to sign up for an organ donor card: Possible predictors and points for intervention.

The KAP model proposes that as knowledge accumulates in a health practice domain, changes in attitude ensue, gradually bringing about change in practices. Our study finds higher scores on knowledge parameters to be positively correlated with attitude scores, r_s_ (298) = .247, *p* < .001, and finds both knowledge and attitude scores to be significantly associated with the intent to practice, *p* = .006 and < .001, respectively. (Refer 3.2.2 and 3.4) According to the knowledge and attitude gaps identified by our study, a three-pronged strategy with a focus on health education, rectifying the common mans’ relationship with the health-care system, and conducive legislative measures is proposed as possible points of intervention to improve organ donation rates is shown in Fig 3.

First, health education in organ donation should be targeted towards: (a) school-going students, who can further educate their family members, a step central to obtaining family support, (b) key-members of society who have the ability to positively influence organ donation in the community, e.g. Imams in the case of Muslim communities, and (c) health-care professionals who should be encouraged to be pro-active in advocating organ donation and addressing patients’ apprehensions. (Refer 4.1.1) Second, continuing medical education and modifications to medical curricula with an emphasis on communication is key to improving patients’ relationships with their doctors or health-care providers. This positive relationship is also required for family support. (Refer 4.2) Third, legislative measures to include provisions for: (a) a legalized and well-regulated system for commercial organ donor-ship, by some measure of financial compensation (e.g. reimbursement, direct payment) or other ‘moral’ incentives in order to protect the rights of the poor, (b) a *legally endorsed* organ donor card, which ensures that those who express their willingness to donate should be able to do so even in the event of non-consent by the deceased donor’s family. This is imperative, as unlike other western countries, the prevalent donor cards in India merely reflect the individuals’ willingness towards deceased organ donation, with the onus of consent falling on the family.[34]

It is important to note both the positive and negative roles of mass and social media platforms in influencing donor registrations. It can aid in spreading awareness and swaying public opinions, but may also hamper the same, through negative media coverage regarding doctors and health-care personnel.

### 4.4 Strengths and limitations of the study

The study’s strength lies in its’ novelty that it is the first in India to explore the knowledge, attitude, and practice regarding organ donation amongst a cohort of drivers. Additionally, the study ensured that we assessed the participants’ actual knowledge rather than perceived knowledge. The study was also conducted in an all-male, educationally, ethnically and socio-economically homogenous group, with a relatively large sample size.

In addition to the inherent limitations of a cross-sectional study, the nature of the orally administered questionnaire could have led to a yes-saying bias,[40] overestimating our outcome variable. This could have been compounded by the fact that our primary outcome measured the intent to practice organ donation i.e. participants’ willingness to sign-up for an organ donor card, rather than the outcome of a tangible increase in registered organ donors.

## Conclusion

Our findings show that nearly half our study cohort had unsatisfactory knowledge and attitudes. However, there were significantly positive correlations between increased knowledge and positive attitudes on the intent to practice organ donation. Therefore, targeted health education, behaviour change communication, and legal interventions, in conjunction, are key to improving organ donor registrations.

## Supporting information

S1 File Chi-square analyses of the participants’ sociodemographic factors and their apprehensions towards organ donation. (PDF)

S1 Table Survey questions, and absolute and relative (%) number of expected responses i.e. correct, affirmative, or positive responses, on knowledge and attitude, regarding organ donation. (DOCX)

S2 Table Survey questions, and correct, affirmative, or positive responses on knowledge, attitude, and practices regarding organ donation with a detailed breakdown of scores or points. (DOCX)

S3 Table Analysis of the outliers excluded from the logistic regression model for the predictors of willingness to sign up for an organ-donor card. ^a^ Unfavourable responses on knowledge parameters; ^b^ Unfavourable responses on attitude parameters; ^c^ Calculated when all 292 responses were included in the regression model.
(DOCX)
