# Supplementary material for: Devising focused strategies to improve organ donor registrations: A cross-sectional study among professional drivers in coastal South India
Source: PLoS One. 2018 Dec 21;13(12):e0209686. doi: 10.1371/journal.pone.0209686 (PMC6303053; doi:10.1371/journal.pone.0209686)
Supplement: S1 File — (PDF) [file pone.0209686.s001.pdf]

S1 File. Chi-Square analyses of the participants' sociodemographic factors and their apprehensions towards organ donation.

Notes

Syntax		CROSSTABS<br><br>/TABLES=Age_participants Educational_status Place_of_residence BY Apprehension_1 Apprehension_2 Apprehension_3 Apprehension_4 Apprehension_5 Apprehension_6 Apprehension_7<br>/FORMAT=DVALUE TABLES<br>/STATISTICS=CHISQ<br>/CELLS=COUNT ROW COLUMN<br>/COUNT ROUND CELL.
Resources	Processor Time	00:00:00.10
	Elapsed Time	00:00:00.00
	Dimensions Requested	2
	Cells Available	524245

Case Processing Summary

	Cases					
	Valid		Missing		Total	
	N	Percent	N	Percent	N	Percent
Age_participants * I feel like I am too old to donate my organs:	292	100.0%	0	0.0%	292	100.0%
Age_participants * I feel my medical co-morbidities prevent me from donating my organs:	292	100.0%	0	0.0%	292	100.0%
Age_participants * I feel the surgery for donating organs will disfigure my body:	292	100.0%	0	0.0%	292	100.0%
Age_participants * I feel my family won't support my decision to donate my organs:	292	100.0%	0	0.0%	292	100.0%
Age_participants * I have concerns that my organs will be used for medical research rather than for patients:	292	100.0%	0	0.0%	292	100.0%

Case Processing Summary

	Cases					
	Valid		Missing		Total	
	N	Percent	N	Percent	N	Percent
Age_participants * I have concerns that my organs will not go to those patients who need it most:	292	100.0%	0	0.0%	292	100.0%
Age_participants * My religious beliefs do not permit me to donate my organs	292	100.0%	0	0.0%	292	100.0%
Educational_status * I feel like I am too old to donate my organs:	292	100.0%	0	0.0%	292	100.0%
Educational_status * I feel my medical co-morbidities prevent me from donating my organs:	292	100.0%	0	0.0%	292	100.0%
Educational_status * I feel the surgery for donating organs will disfigure my body:	292	100.0%	0	0.0%	292	100.0%
Educational_status * I feel my family won't support my decision to donate my organs:	292	100.0%	0	0.0%	292	100.0%
Educational_status * I have concerns that my organs will be used for medical research rather than for patients:	292	100.0%	0	0.0%	292	100.0%
Educational_status * I have concerns that my organs will not go to those patients who need it most:	292	100.0%	0	0.0%	292	100.0%
Educational_status * My religious beliefs do not permit me to donate my organs	292	100.0%	0	0.0%	292	100.0%
Place_of_residence * I feel like I am too old to donate my organs:	292	100.0%	0	0.0%	292	100.0%
Place_of_residence * I feel my medical co-morbidities prevent me from donating my organs:	292	100.0%	0	0.0%	292	100.0%

Case Processing Summary

	Cases					
	Valid		Missing		Total	
	N	Percent	N	Percent	N	Percent
Place_of_residence * I feel the surgery for donating organs will disfigure my body:	292	100.0%	0	0.0%	292	100.0%
Place_of_residence * I feel my family won't support my decision to donate my organs:	292	100.0%	0	0.0%	292	100.0%
Place_of_residence * I have concerns that my organs will be used for medical research rather than for patients:	292	100.0%	0	0.0%	292	100.0%
Place_of_residence * I have concerns that my organs will not go to those patients who need it most:	292	100.0%	0	0.0%	292	100.0%
Place_of_residence * My religious beliefs do not permit me to donate my organs	292	100.0%	0	0.0%	292	100.0%

Age_participants * I feel like I am too old to donate my organs:

Crosstab

			I feel like I am too old to donate my organs: No, I do not have such feelings.	Yes
Age_participants	60 - 73	Count	13	0
		% within Age_participants	100.0%	0.0%
		% within I feel like I am too old to donate my organs:	4.6%	0.0%
	50 - 59	Count	35	3
		% within Age_participants	92.1%	7.9%
		% within I feel like I am too old to donate my organs:	12.5%	27.3%
	40 - 49	Count	69	5
		% within Age_participants	93.2%	6.8%
		% within I feel like I am too old to donate my organs:	24.6%	45.5%
	30 - 39	Count	90	2
		% within Age_participants	97.8%	2.2%
		% within I feel like I am too old to donate my organs:	32.0%	18.2%
	19 - 29	Count	74	1
		% within Age_participants	98.7%	1.3%
		% within I feel like I am too old to donate my organs:	26.3%	9.1%
Total		Count	281	11
		% within Age_participants	96.2%	3.8%
		% within I feel like I am too old to donate my organs:	100.0%	100.0%

Crosstab

			Total
Age_participants	60 - 73	Count	13
		% within Age_participants	100.0%
		% within I feel like I am too old to donate my organs:	4.5%
	50 - 59	Count	38
		% within Age_participants	100.0%
		% within I feel like I am too old to donate my organs:	13.0%
	40 - 49	Count	74
		% within Age_participants	100.0%
		% within I feel like I am too old to donate my organs:	25.3%
	30 - 39	Count	92
		% within Age_participants	100.0%
		% within I feel like I am too old to donate my organs:	31.5%
	19 - 29	Count	75
		% within Age_participants	100.0%
		% within I feel like I am too old to donate my organs:	25.7%
Total		Count	292
		% within Age_participants	100.0%
		% within I feel like I am too old to donate my organs:	100.0%

Chi-Square Tests

	Value	df	Asymptotic Significance (2-sided)
Pearson Chi-Square	5.989[a]	4	.200
Likelihood Ratio	6.232	4	.182
Linear-by-Linear Association	2.396	1	.122
N of Valid Cases	292		

a. 5 cells (50.0%) have expected count less than 5. The minimum expected count is .49.

Age_participants * I feel my medical co-morbidities prevent me from donating my organs:

Crosstab

			I feel my medical co-morbidities prevent me from donating my organs:	
			No, I do not have such feelings.	Yes
Age_participants	60 - 73	Count	12	1
		% within Age_participants	92.3%	7.7%
		% within I feel my medical co-morbidities prevent me from donating my organs:	4.3%	7.1%
	50 - 59	Count	36	2
		% within Age_participants	94.7%	5.3%
		% within I feel my medical co-morbidities prevent me from donating my organs:	12.9%	14.3%
	40 - 49	Count	71	3
		% within Age_participants	95.9%	4.1%
		% within I feel my medical co-morbidities prevent me from donating my organs:	25.5%	21.4%
	30 - 39	Count	87	5
		% within Age_participants	94.6%	5.4%
		% within I feel my medical co-morbidities prevent me from donating my organs:	31.3%	35.7%

Crosstab

			Total
Age_participants	60 - 73	Count	13
		% within Age_participants	100.0%
		% within I feel my medical co-morbidities prevent me from donating my organs:	4.5%
	50 - 59	Count	38
		% within Age_participants	100.0%
		% within I feel my medical co-morbidities prevent me from donating my organs:	13.0%
	40 - 49	Count	74
		% within Age_participants	100.0%
		% within I feel my medical co-morbidities prevent me from donating my organs:	25.3%
	30 - 39	Count	92
		% within Age_participants	100.0%
		% within I feel my medical co-morbidities prevent me from donating my organs:	31.5%

Crosstab

		I feel my medical co-morbidities prevent me from donating my organs: No, I do not have such feelings.	Yes
19 - 29	Count	72	3
	% within Age_participants	96.0%	4.0%
	% within I feel my medical co-morbidities prevent me from donating my organs:	25.9%	21.4%
Total	Count	278	14
	% within Age_participants	95.2%	4.8%
	% within I feel my medical co-morbidities prevent me from donating my organs:	100.0%	100.0%

Crosstab

		Total
19 - 29	Count	75
	% within Age_participants	100.0%
	% within I feel my medical co-morbidities prevent me from donating my organs:	25.7%
Total	Count	292
	% within Age_participants	100.0%
	% within I feel my medical co-morbidities prevent me from donating my organs:	100.0%

Chi-Square Tests

	Value	df	Asymptotic Significance (2-sided)
Pearson Chi-Square	.533[a]	4	.970
Likelihood Ratio	.504	4	.973
Linear-by-Linear Association	.138	1	.711
N of Valid Cases	292		

a. 5 cells (50.0%) have expected count less than 5. The minimum expected count is .62.

Age_participants * I feel the surgery for donating organs will di sfigure my body:

Crosstab

			I feel the surgery for donating organs will disfigure my body:	
			No, I do not have such feelings.	Yes
Age_participants	60 - 73	Count	13	0
		% within Age_participants	100.0%	0.0%
		% within I feel the surgery for donating organs will disfigure my body:	4.7%	0.0%
	50 - 59	Count	37	1
		% within Age_participants	97.4%	2.6%
		% within I feel the surgery for donating organs will disfigure my body:	13.5%	5.9%
	40 - 49	Count	69	5
		% within Age_participants	93.2%	6.8%
		% within I feel the surgery for donating organs will disfigure my body:	25.1%	29.4%
	30 - 39	Count	87	5
		% within Age_participants	94.6%	5.4%
		% within I feel the surgery for donating organs will disfigure my body:	31.6%	29.4%

Crosstab

			Total
Age_participants	60 - 73	Count	13
		% within Age_participants	100.0%
		% within I feel the surgery for donating organs will disfigure my body:	4.5%
	50 - 59	Count	38
		% within Age_participants	100.0%
		% within I feel the surgery for donating organs will disfigure my body:	13.0%
	40 - 49	Count	74
		% within Age_participants	100.0%
		% within I feel the surgery for donating organs will disfigure my body:	25.3%
	30 - 39	Count	92
		% within Age_participants	100.0%
		% within I feel the surgery for donating organs will disfigure my body:	31.5%

Crosstab

		I feel the surgery for donating organs will disfigure my body: No, I do not have such feelings.	Yes
19 - 29	Count	69	6
	% within Age_participants	92.0%	8.0%
	% within I feel the surgery for donating organs will disfigure my body:	25.1%	35.3%
Total	Count	275	17
	% within Age_participants	94.2%	5.8%
	% within I feel the surgery for donating organs will disfigure my body:	100.0%	100.0%

Crosstab

		Total
19 - 29	Count	75
	% within Age_participants	100.0%
	% within I feel the surgery for donating organs will disfigure my body:	25.7%
Total	Count	292
	% within Age_participants	100.0%
	% within I feel the surgery for donating organs will disfigure my body:	100.0%

Chi-Square Tests

	Value	df	Asymptotic Significance (2-sided)
Pearson Chi-Square	2.301[a]	4	.681
Likelihood Ratio	3.160	4	.531
Linear-by-Linear Association	1.546	1	.214
N of Valid Cases	292		

a. 4 cells (40.0%) have expected count less than 5. The minimum expected count is .76.

Age_participants * I feel my family won’t support my decision to donate my organs:

Crosstab

			I feel my family won’t support my decision to donate my organs:	
			no / NA	yes
Age_participants	60 - 73	Count	12	1
		% within Age_participants	92.3%	7.7%
		% within I feel my family won’t support my decision to donate my organs:	4.8%	2.4%
	50 - 59	Count	32	6
		% within Age_participants	84.2%	15.8%
		% within I feel my family won’t support my decision to donate my organs:	12.8%	14.3%
	40 - 49	Count	64	10
		% within Age_participants	86.5%	13.5%
		% within I feel my family won’t support my decision to donate my organs:	25.6%	23.8%
	30 - 39	Count	81	11
		% within Age_participants	88.0%	12.0%
		% within I feel my family won’t support my decision to donate my organs:	32.4%	26.2%

Crosstab

			Total
Age_participants	60 - 73	Count	13
		% within Age_participants	100.0%
		% within I feel my family won't support my decision to donate my organs:	4.5%
	50 - 59	Count	38
		% within Age_participants	100.0%
		% within I feel my family won't support my decision to donate my organs:	13.0%
	40 - 49	Count	74
		% within Age_participants	100.0%
		% within I feel my family won't support my decision to donate my organs:	25.3%
	30 - 39	Count	92
		% within Age_participants	100.0%
		% within I feel my family won't support my decision to donate my organs:	31.5%

Crosstab

		I feel my family won’t support my decision to donate my organs: no / NA	yes
19 - 29	Count	61	14
	% within Age_participants	81.3%	18.7%
	% within I feel my family won’t support my decision to donate my organs:	24.4%	33.3%
Total	Count	250	42
	% within Age_participants	85.6%	14.4%
	% within I feel my family won’t support my decision to donate my organs:	100.0%	100.0%

Crosstab

		Total
19 - 29	Count	75
	% within Age_participants	100.0%
	% within I feel my family won’t support my decision to donate my organs:	25.7%
Total	Count	292
	% within Age_participants	100.0%
	% within I feel my family won’t support my decision to donate my organs:	100.0%

Chi-Square Tests

	Value	df	Asymptotic Significance (2-sided)
Pearson Chi-Square	2.136[a]	4	.711
Likelihood Ratio	2.160	4	.706
Linear-by-Linear Association	.631	1	.427
N of Valid Cases	292		

a. 1 cells (10.0%) have expected count less than 5. The minimum expected count is 1.87.

Age_participants * I have concerns that my organs will be used for medical research rather than for patients:

Crosstab

			I have concerns that my organs will be used for medical research rather than for patients:	
			No, I do not have any such concerns.	Yes
Age_participants	60 - 73	Count	11	2
		% within Age_participants	84.6%	15.4%
		% within I have concerns that my organs will be used for medical research rather than for patients:	4.7%	3.4%
	50 - 59	Count	31	7
		% within Age_participants	81.6%	18.4%
		% within I have concerns that my organs will be used for medical research rather than for patients:	13.2%	12.1%
	40 - 49	Count	55	19
		% within Age_participants	74.3%	25.7%
		% within I have concerns that my organs will be used for medical research rather than for patients:	23.5%	32.8%

Crosstab

			Total
Age_participants	60 - 73	Count	13
		% within Age_participants	100.0%
		% within I have concerns that my organs will be used for medical research rather than for patients:	4.5%
	50 - 59	Count	38
		% within Age_participants	100.0%
		% within I have concerns that my organs will be used for medical research rather than for patients:	13.0%
	40 - 49	Count	74
		% within Age_participants	100.0%
		% within I have concerns that my organs will be used for medical research rather than for patients:	25.3%

Crosstab

		I have concerns that my organs will be used for medical research rather than for patients: No, I do not have any such concerns.	Yes
30 - 39	Count	78	14
	% within Age_participants	84.8%	15.2%
	% within I have concerns that my organs will be used for medical research rather than for patients:	33.3%	24.1%
19 - 29	Count	59	16
	% within Age_participants	78.7%	21.3%
	% within I have concerns that my organs will be used for medical research rather than for patients:	25.2%	27.6%
Total	Count	234	58
	% within Age_participants	80.1%	19.9%
	% within I have concerns that my organs will be used for medical research rather than for patients:	100.0%	100.0%

Crosstab

			Total
	30 - 39	Count	92
		% within Age_participants	100.0%
		% within I have concerns that my organs will be used for medical research rather than for patients:	31.5%
	19 - 29	Count	75
		% within Age_participants	100.0%
		% within I have concerns that my organs will be used for medical research rather than for patients:	25.7%
Total		Count	292
		% within Age_participants	100.0%
		% within I have concerns that my organs will be used for medical research rather than for patients:	100.0%

Chi-Square Tests

	Value	df	Asymptotic Significance (2-sided)
Pearson Chi-Square	3.133[a]	4	.536
Likelihood Ratio	3.127	4	.537
Linear-by-Linear Association	.002	1	.963
N of Valid Cases	292		

a. 1 cells (10.0%) have expected count less than 5. The minimum expected count is 2.58.

Age_participants * I have concerns that my organs will not go to those patients who need it most:

Crosstab

			I have concerns that my organs will not go to those patients who need it most:	
			No, I do not have any such concerns.	Yes
Age_participants	60 - 73	Count	10	3
		% within Age_participants	76.9%	23.1%
		% within I have concerns that my organs will not go to those patients who need it most:	4.6%	4.1%
	50 - 59	Count	30	8
		% within Age_participants	78.9%	21.1%
		% within I have concerns that my organs will not go to those patients who need it most:	13.7%	11.0%
	40 - 49	Count	52	22
		% within Age_participants	70.3%	29.7%
		% within I have concerns that my organs will not go to those patients who need it most:	23.7%	30.1%
	30 - 39	Count	71	21
		% within Age_participants	77.2%	22.8%
		% within I have concerns that my organs will not go to those patients who need it most:	32.4%	28.8%
	19 - 29	Count	56	19
		% within Age_participants	74.7%	25.3%
		% within I have concerns that my organs will not go to those patients who need it most:	25.6%	26.0%
Total		Count	219	73
		% within Age_participants	75.0%	25.0%
		% within I have concerns that my organs will not go to those patients who need it most:	100.0%	100.0%

Crosstab

			Total
Age_participants	60 - 73	Count	13
		% within Age_participants	100.0%
		% within I have concerns that my organs will not go to those patients who need it most:	4.5%
	50 - 59	Count	38
		% within Age_participants	100.0%
		% within I have concerns that my organs will not go to those patients who need it most:	13.0%
	40 - 49	Count	74
		% within Age_participants	100.0%
		% within I have concerns that my organs will not go to those patients who need it most:	25.3%
	30 - 39	Count	92
		% within Age_participants	100.0%
		% within I have concerns that my organs will not go to those patients who need it most:	31.5%
	19 - 29	Count	75
		% within Age_participants	100.0%
		% within I have concerns that my organs will not go to those patients who need it most:	25.7%
Total		Count	292
		% within Age_participants	100.0%
		% within I have concerns that my organs will not go to those patients who need it most:	100.0%

Chi-Square Tests

	Value	df	Asymptotic Significance (2-sided)
Pearson Chi-Square	1.461[a]	4	.834
Likelihood Ratio	1.445	4	.836
Linear-by-Linear Association	.004	1	.952
N of Valid Cases	292		

a. 1 cells (10.0%) have expected count less than 5. The minimum expected count is 3.25.

Age_participants * My religious beliefs do not permit me to don ate my organs

Crosstab

			My religious beliefs do not permit me to donate my organs	
			No, I have no such religious restrictions.	Yes
Age_participants	60 - 73	Count	13	0
		% within Age_participants	100.0%	0.0%
		% within My religious beliefs do not permit me to donate my organs	4.6%	0.0%
	50 - 59	Count	36	2
		% within Age_participants	94.7%	5.3%
		% within My religious beliefs do not permit me to donate my organs	12.8%	20.0%
	40 - 49	Count	71	3
		% within Age_participants	95.9%	4.1%
		% within My religious beliefs do not permit me to donate my organs	25.2%	30.0%
	30 - 39	Count	90	2
		% within Age_participants	97.8%	2.2%
		% within My religious beliefs do not permit me to donate my organs	31.9%	20.0%

Crosstab

			Total
Age_participants	60 - 73	Count	13
		% within Age_participants	100.0%
		% within My religious beliefs do not permit me to donate my organs	4.5%
	50 - 59	Count	38
		% within Age_participants	100.0%
		% within My religious beliefs do not permit me to donate my organs	13.0%
	40 - 49	Count	74
		% within Age_participants	100.0%
		% within My religious beliefs do not permit me to donate my organs	25.3%
	30 - 39	Count	92
		% within Age_participants	100.0%
		% within My religious beliefs do not permit me to donate my organs	31.5%

Crosstab

		My religious beliefs do not permit me to donate my organs	
		No, I have no such religious restrictions.	Yes
19 - 29	Count	72	3
	% within Age_participants	96.0%	4.0%
	% within My religious beliefs do not permit me to donate my organs	25.5%	30.0%
Total	Count	282	10
	% within Age_participants	96.6%	3.4%
	% within My religious beliefs do not permit me to donate my organs	100.0%	100.0%

Crosstab

		Total
19 - 29	Count	75
	% within Age_participants	100.0%
	% within My religious beliefs do not permit me to donate my organs	25.7%
Total	Count	292
	% within Age_participants	100.0%
	% within My religious beliefs do not permit me to donate my organs	100.0%

Chi-Square Tests

	Value	df	Asymptotic Significance (2-sided)
Pearson Chi-Square	1.448[a]	4	.836
Likelihood Ratio	1.895	4	.755
Linear-by-Linear Association	.001	1	.978
N of Valid Cases	292		

a. 5 cells (50.0%) have expected count less than 5. The minimum expected count is .45.

Educational_status * I feel like I am too old to donate my organs:

Crosstab

			I feel like I am too old to...
			No, I do not have such feelings.
Educational_status	Graduate degree / higher	Count	29
		% within Educational_status	100.0%
		% within I feel like I am too old to donate my organs:	10.3%
	PUC (11th or 12th class)	Count	64
		% within Educational_status	98.5%
		% within I feel like I am too old to donate my organs:	22.8%
	Middle & High School (upto 10th class)	Count	168
		% within Educational_status	96.0%
		% within I feel like I am too old to donate my organs:	59.8%

Crosstab

			I feel like I am too old to... Yes
Educational_status	Graduate degree / higher	Count	0
		% within Educational_status	0.0%
		% within I feel like I am too old to donate my organs:	0.0%
	PUC (11th or 12th class)	Count	1
		% within Educational_status	1.5%
		% within I feel like I am too old to donate my organs:	9.1%
	Middle & High School (upto 10th class)	Count	7
		% within Educational_status	4.0%
		% within I feel like I am too old to donate my organs:	63.6%

Crosstab

			Total
Educational_status	Graduate degree / higher	Count	29
		% within Educational_status	100.0%
		% within I feel like I am too old to donate my organs:	9.9%
	PUC (11th or 12th class)	Count	65
		% within Educational_status	100.0%
		% within I feel like I am too old to donate my organs:	22.3%
	Middle & High School (upto 10th class)	Count	175
		% within Educational_status	100.0%
		% within I feel like I am too old to donate my organs:	59.9%

Crosstab

		I feel like I am too old to...
		No, I do not have such feelings.
Illiterate & Primary School	Count	20
	% within Educational_status	87.0%
	% within I feel like I am too old to donate my organs:	7.1%
Total	Count	281
	% within Educational_status	96.2%
	% within I feel like I am too old to donate my organs:	100.0%

Crosstab

		I feel like I am too old to...
		Yes
Illiterate & Primary School	Count	3
	% within Educational_status	13.0%
	% within I feel like I am too old to donate my organs:	27.3%
Total	Count	11
	% within Educational_status	3.8%
	% within I feel like I am too old to donate my organs:	100.0%

Crosstab

		Total
Illiterate & Primary School	Count	23
	% within Educational_status	100.0%
	% within I feel like I am too old to donate my organs:	7.9%
Total	Count	292
	% within Educational_status	100.0%
	% within I feel like I am too old to donate my organs:	100.0%

Chi-Square Tests

	Value	df	Asymptotic Significance (2-sided)
Pearson Chi-Square	7.511[a]	3	.057
Likelihood Ratio	6.790	3	.079
Linear-by-Linear Association	5.386	1	.020
N of Valid Cases	292		

a. 3 cells (37.5%) have expected count less than 5. The minimum expected count is .87.

Educational_status * I feel my medical co-morbidities prevent me from donating my organs:

Crosstab

			I feel my medical co-morbidities ...
			No, I do not have such feelings.
Educational_status	Graduate degree / higher	Count	29
		% within Educational_status	100.0%
		% within I feel my medical co-morbidities prevent me from donating my organs:	10.4%
	PUC (11th or 12th class)	Count	63
		% within Educational_status	96.9%
		% within I feel my medical co-morbidities prevent me from donating my organs:	22.7%
	Middle & High School (upto 10th class)	Count	165
		% within Educational_status	94.3%
		% within I feel my medical co-morbidities prevent me from donating my organs:	59.4%
	Illiterate & Primary School	Count	21
		% within Educational_status	91.3%
		% within I feel my medical co-morbidities prevent me from donating my organs:	7.6%
Total		Count	278
		% within Educational_status	95.2%
		% within I feel my medical co-morbidities prevent me from donating my organs:	100.0%

Crosstab

			I feel my medical co-morbidities ...
			Yes
Educational_status	Graduate degree / higher	Count	0
		% within Educational_status	0.0%
		% within I feel my medical co-morbidities prevent me from donating my organs:	0.0%
	PUC (11th or 12th class)	Count	2
		% within Educational_status	3.1%
		% within I feel my medical co-morbidities prevent me from donating my organs:	14.3%
	Middle & High School (upto 10th class)	Count	10
		% within Educational_status	5.7%
		% within I feel my medical co-morbidities prevent me from donating my organs:	71.4%
	Illiterate & Primary School	Count	2
		% within Educational_status	8.7%
		% within I feel my medical co-morbidities prevent me from donating my organs:	14.3%
Total		Count	14
		% within Educational_status	4.8%
		% within I feel my medical co-morbidities prevent me from donating my organs:	100.0%

Crosstab

			Total
Educational_status	Graduate degree / higher	Count	29
		% within Educational_status	100.0%
		% within I feel my medical co-morbidities prevent me from donating my organs:	9.9%
	PUC (11th or 12th class)	Count	65
		% within Educational_status	100.0%
		% within I feel my medical co-morbidities prevent me from donating my organs:	22.3%
	Middle & High School (upto 10th class)	Count	175
		% within Educational_status	100.0%
		% within I feel my medical co-morbidities prevent me from donating my organs:	59.9%
	Illiterate & Primary School	Count	23
		% within Educational_status	100.0%
		% within I feel my medical co-morbidities prevent me from donating my organs:	7.9%
Total		Count	292
		% within Educational_status	100.0%
		% within I feel my medical co-morbidities prevent me from donating my organs:	100.0%

Chi-Square Tests

	Value	df	Asymptotic Significance (2-sided)
Pearson Chi-Square	2.972[a]	3	.396
Likelihood Ratio	4.259	3	.235
Linear-by-Linear Association	2.956	1	.086
N of Valid Cases	292		

a. 3 cells (37.5%) have expected count less than 5. The minimum expected count is 1.10.

Educational_status * I feel the surgery for donating organs will disfigure my body:

Crosstab

			I feel the surgery for ...
			No, I do not have such feelings.
Educational_status	Graduate degree / higher	Count	28
		% within Educational_status	96.6%
		% within I feel the surgery for donating organs will disfigure my body:	10.2%
	PUC (11th or 12th class)	Count	57
		% within Educational_status	87.7%
		% within I feel the surgery for donating organs will disfigure my body:	20.7%
	Middle & High School (upto 10th class)	Count	168
		% within Educational_status	96.0%
		% within I feel the surgery for donating organs will disfigure my body:	61.1%
	Illiterate & Primary School	Count	22
		% within Educational_status	95.7%
		% within I feel the surgery for donating organs will disfigure my body:	8.0%

Crosstab

			I feel the surgery for ...
			Yes
Educational_status	Graduate degree / higher	Count	1
		% within Educational_status	3.4%
		% within I feel the surgery for donating organs will disfigure my body:	5.9%
	PUC (11th or 12th class)	Count	8
		% within Educational_status	12.3%
		% within I feel the surgery for donating organs will disfigure my body:	47.1%
	Middle & High School (upto 10th class)	Count	7
		% within Educational_status	4.0%
		% within I feel the surgery for donating organs will disfigure my body:	41.2%
	Illiterate & Primary School	Count	1
		% within Educational_status	4.3%
		% within I feel the surgery for donating organs will disfigure my body:	5.9%

Crosstab

			Total
Educational_status	Graduate degree / higher	Count	29
		% within Educational_status	100.0%
		% within I feel the surgery for donating organs will disfigure my body:	9.9%
	PUC (11th or 12th class)	Count	65
		% within Educational_status	100.0%
		% within I feel the surgery for donating organs will disfigure my body:	22.3%
	Middle & High School (upto 10th class)	Count	175
		% within Educational_status	100.0%
		% within I feel the surgery for donating organs will disfigure my body:	59.9%
	Illiterate & Primary School	Count	23
		% within Educational_status	100.0%
		% within I feel the surgery for donating organs will disfigure my body:	7.9%

Crosstab

		I feel the surgery for ...
		No, I do not have such feelings.
Total	Count	275
	% within Educational_status	94.2%
	% within I feel the surgery for donating organs will disfigure my body:	100.0%

Crosstab

		I feel the surgery for ... Yes
Total	Count	17
	% within Educational_status	5.8%
	% within I feel the surgery for donating organs will disfigure my body:	100.0%

Crosstab

		Total
Total	Count	292
	% within Educational_status	100.0%
	% within I feel the surgery for donating organs will disfigure my body:	100.0%

Chi-Square Tests

	Value	df	Asymptotic Significance (2-sided)
Pearson Chi-Square	6.435[a]	3	.092
Likelihood Ratio	5.472	3	.140
Linear-by-Linear Association	1.081	1	.298
N of Valid Cases	292		

a. 3 cells (37.5%) have expected count less than 5. The minimum expected count is 1.34.

Educational_status * I feel my family won’t support my decision to donate my organs:

Crosstab

			I feel my family won't support my decision to.
			no / NA
Educational_status	Graduate degree / higher	Count	27
		% within Educational_status	93.1%
		% within I feel my family won't support my decision to donate my organs:	10.8%
	PUC (11th or 12th class)	Count	53
		% within Educational_status	81.5%
		% within I feel my family won't support my decision to donate my organs:	21.2%
	Middle & High School (upto 10th class)	Count	150
		% within Educational_status	85.7%
		% within I feel my family won't support my decision to donate my organs:	60.0%
	Illiterate & Primary School	Count	20
		% within Educational_status	87.0%
		% within I feel my family won't support my decision to donate my organs:	8.0%
Total		Count	250
		% within Educational_status	85.6%
		% within I feel my family won't support my decision to donate my organs:	100.0%

Crosstab

			I feel my family won't support my decision to..
			yes
Educational_status	Graduate degree / higher	Count	2
		% within Educational_status	6.9%
		% within I feel my family won't support my decision to donate my organs:	4.8%
	PUC (11th or 12th class)	Count	12
		% within Educational_status	18.5%
		% within I feel my family won't support my decision to donate my organs:	28.6%
	Middle & High School (upto 10th class)	Count	25
		% within Educational_status	14.3%
		% within I feel my family won't support my decision to donate my organs:	59.5%
	Illiterate & Primary School	Count	3
		% within Educational_status	13.0%
		% within I feel my family won't support my decision to donate my organs:	7.1%
Total		Count	42
		% within Educational_status	14.4%
		% within I feel my family won't support my decision to donate my organs:	100.0%

Crosstab

			Total
Educational_status	Graduate degree / higher	Count	29
		% within Educational_status	100.0%
		% within I feel my family won't support my decision to donate my organs:	9.9%
	PUC (11th or 12th class)	Count	65
		% within Educational_status	100.0%
		% within I feel my family won't support my decision to donate my organs:	22.3%
	Middle & High School (upto 10th class)	Count	175
		% within Educational_status	100.0%
		% within I feel my family won't support my decision to donate my organs:	59.9%
	Illiterate & Primary School	Count	23
		% within Educational_status	100.0%
		% within I feel my family won't support my decision to donate my organs:	7.9%
Total		Count	292
		% within Educational_status	100.0%
		% within I feel my family won't support my decision to donate my organs:	100.0%

Chi-Square Tests

	Value	df	Asymptotic Significance (2-sided)
Pearson Chi-Square	2.233[a]	3	.526
Likelihood Ratio	2.440	3	.486
Linear-by-Linear Association	.091	1	.763
N of Valid Cases	292		

a. 2 cells (25.0%) have expected count less than 5. The minimum expected count is 3.31.

Educational_status * I have concerns that my organs will be use d for medical research rather than for patients:

Crosstab

			I have concerns that my organs will be used for ...
			No, I do not have any such concerns.
Educational_status	Graduate degree / higher	Count	26
		% within Educational_status	89.7%
		% within I have concerns that my organs will be used for medical research rather than for patients:	11.1%
	PUC (11th or 12th class)	Count	50
		% within Educational_status	76.9%
		% within I have concerns that my organs will be used for medical research rather than for patients:	21.4%
	Middle & High School (upto 10th class)	Count	142
		% within Educational_status	81.1%
		% within I have concerns that my organs will be used for medical research rather than for patients:	60.7%

Crosstab

			I have concerns that my organs will be used for medical ...
			Yes
Educational_status	Graduate degree / higher	Count	3
		% within Educational_status	10.3%
		% within I have concerns that my organs will be used for medical research rather than for patients:	5.2%
	PUC (11th or 12th class)	Count	15
		% within Educational_status	23.1%
		% within I have concerns that my organs will be used for medical research rather than for patients:	25.9%
	Middle & High School (upto 10th class)	Count	33
		% within Educational_status	18.9%
		% within I have concerns that my organs will be used for medical research rather than for patients:	56.9%

Crosstab

			Total
Educational_status	Graduate degree / higher	Count	29
		% within Educational_status	100.0%
		% within I have concerns that my organs will be used for medical research rather than for patients:	9.9%
	PUC (11th or 12th class)	Count	65
		% within Educational_status	100.0%
		% within I have concerns that my organs will be used for medical research rather than for patients:	22.3%
	Middle & High School (upto 10th class)	Count	175
		% within Educational_status	100.0%
		% within I have concerns that my organs will be used for medical research rather than for patients:	59.9%

Crosstab

		I have concerns that my organs will be used for ...
		No, I do not have any such concerns.
Illiterate & Primary School	Count	16
	% within Educational_status	69.6%
	% within I have concerns that my organs will be used for medical research rather than for patients:	6.8%
Total	Count	234
	% within Educational_status	80.1%
	% within I have concerns that my organs will be used for medical research rather than for patients:	100.0%

Crosstab

		I have concerns that my organs will be used for medical ...
		Yes
Illiterate & Primary School	Count	7
	% within Educational_status	30.4%
	% within I have concerns that my organs will be used for medical research rather than for patients:	12.1%
Total	Count	58
	% within Educational_status	19.9%
	% within I have concerns that my organs will be used for medical research rather than for patients:	100.0%

Crosstab

		Total
Illiterate & Primary School	Count	23
	% within Educational_status	100.0%
	% within I have concerns that my organs will be used for medical research rather than for patients:	7.9%
Total	Count	292
	% within Educational_status	100.0%
	% within I have concerns that my organs will be used for medical research rather than for patients:	100.0%

Chi-Square Tests

	Value	df	Asymptotic Significance (2-sided)
Pearson Chi-Square	3.798[a]	3	.284
Likelihood Ratio	3.888	3	.274
Linear-by-Linear Association	1.268	1	.260
N of Valid Cases	292		

a. 1 cells (12.5%) have expected count less than 5. The minimum expected count is 4.57.

Educational_status * I have concerns that my organs will not go to those patients who need it most:

Crosstab

			I have concerns that my organs will.
			No, I do not have any such concerns.
Educational_status	Graduate degree / higher	Count	24
		% within Educational_status	82.8%
		% within I have concerns that my organs will not go to those patients who need it most:	11.0%
	PUC (11th or 12th class)	Count	52
		% within Educational_status	80.0%
		% within I have concerns that my organs will not go to those patients who need it most:	23.7%
	Middle & High School (upto 10th class)	Count	127
		% within Educational_status	72.6%
		% within I have concerns that my organs will not go to those patients who need it most:	58.0%
	Illiterate & Primary School	Count	16
		% within Educational_status	69.6%
		% within I have concerns that my organs will not go to those patients who need it most:	7.3%
Total		Count	219
		% within Educational_status	75.0%
		% within I have concerns that my organs will not go to those patients who need it most:	100.0%

Crosstab

			I have concerns that my organs will not go to...
			Yes
Educational_status	Graduate degree / higher	Count	5
		% within Educational_status	17.2%
		% within I have concerns that my organs will not go to those patients who need it most:	6.8%
	PUC (11th or 12th class)	Count	13
		% within Educational_status	20.0%
		% within I have concerns that my organs will not go to those patients who need it most:	17.8%
	Middle & High School (upto 10th class)	Count	48
		% within Educational_status	27.4%
		% within I have concerns that my organs will not go to those patients who need it most:	65.8%
	Illiterate & Primary School	Count	7
		% within Educational_status	30.4%
		% within I have concerns that my organs will not go to those patients who need it most:	9.6%
Total		Count	73
		% within Educational_status	25.0%
		% within I have concerns that my organs will not go to those patients who need it most:	100.0%

Crosstab

			Total
Educational_status	Graduate degree / higher	Count	29
		% within Educational_status	100.0%
		% within I have concerns that my organs will not go to those patients who need it most:	9.9%
	PUC (11th or 12th class)	Count	65
		% within Educational_status	100.0%
		% within I have concerns that my organs will not go to those patients who need it most:	22.3%
	Middle & High School (upto 10th class)	Count	175
		% within Educational_status	100.0%
		% within I have concerns that my organs will not go to those patients who need it most:	59.9%
	Illiterate & Primary School	Count	23
		% within Educational_status	100.0%
		% within I have concerns that my organs will not go to those patients who need it most:	7.9%
Total		Count	292
		% within Educational_status	100.0%
		% within I have concerns that my organs will not go to those patients who need it most:	100.0%

Chi-Square Tests

	Value	df	Asymptotic Significance (2-sided)
Pearson Chi-Square	2.710[a]	3	.438
Likelihood Ratio	2.806	3	.423
Linear-by-Linear Association	2.536	1	.111
N of Valid Cases	292		

a. 0 cells (0.0%) have expected count less than 5. The minimum expected count is 5.75.

Educational_status * My religious beliefs do not permit me to donate my organs

Crosstab

			My religious beliefs do not permit me to ..
			No, I have no such religious restrictions.
Educational_status	Graduate degree / higher	Count	27
		% within Educational_status	93.1%
		% within My religious beliefs do not permit me to donate my organs	9.6%
	PUC (11th or 12th class)	Count	62
		% within Educational_status	95.4%
		% within My religious beliefs do not permit me to donate my organs	22.0%
	Middle & High School (upto 10th class)	Count	171
		% within Educational_status	97.7%
		% within My religious beliefs do not permit me to donate my organs	60.6%

Crosstab

			My religious beliefs do not permit me to ...
			Yes
Educational_status	Graduate degree / higher	Count	2
		% within Educational_status	6.9%
		% within My religious beliefs do not permit me to donate my organs	20.0%
	PUC (11th or 12th class)	Count	3
		% within Educational_status	4.6%
		% within My religious beliefs do not permit me to donate my organs	30.0%
	Middle & High School (upto 10th class)	Count	4
		% within Educational_status	2.3%
		% within My religious beliefs do not permit me to donate my organs	40.0%

Crosstab

			Total
Educational_status	Graduate degree / higher	Count	29
		% within Educational_status	100.0%
		% within My religious beliefs do not permit me to donate my organs	9.9%
	PUC (11th or 12th class)	Count	65
		% within Educational_status	100.0%
		% within My religious beliefs do not permit me to donate my organs	22.3%
	Middle & High School (upto 10th class)	Count	175
		% within Educational_status	100.0%
		% within My religious beliefs do not permit me to donate my organs	59.9%

Crosstab

			My religious beliefs do not permit me to ..
			No, I have no such religious restrictions.
	Illiterate & Primary School	Count	22
		% within Educational_status	95.7%
		% within My religious beliefs do not permit me to donate my organs	7.8%
Total		Count	282
		% within Educational_status	96.6%
		% within My religious beliefs do not permit me to donate my organs	100.0%

Crosstab

		My religious beliefs do not permit me to ... Yes
Illiterate & Primary School	Count	1
	% within Educational_status	4.3%
	% within My religious beliefs do not permit me to donate my organs	10.0%
Total	Count	10
	% within Educational_status	3.4%
	% within My religious beliefs do not permit me to donate my organs	100.0%

Crosstab

		Total
Illiterate & Primary School	Count	23
	% within Educational_status	100.0%
	% within My religious beliefs do not permit me to donate my organs	7.9%
Total	Count	292
	% within Educational_status	100.0%
	% within My religious beliefs do not permit me to donate my organs	100.0%

Chi-Square Tests

	Value	df	Asymptotic Significance (2-sided)
Pearson Chi-Square	2.081[a]	3	.556
Likelihood Ratio	1.905	3	.592
Linear-by-Linear Association	1.177	1	.278
N of Valid Cases	292		

a. 3 cells (37.5%) have expected count less than 5. The minimum expected count is .79.

Place_of_residence * I feel like I am too old to donate my organs:

Crosstab

			I feel like I am too old to donate my organs:	
			No, I do not have such feelings.	Yes
Place_of_residence	Urban	Count	135	4
		% within Place_of_residence	97.1%	2.9%
		% within I feel like I am too old to donate my organs:	48.0%	36.4%
	Rural	Count	146	7
		% within Place_of_residence	95.4%	4.6%
		% within I feel like I am too old to donate my organs:	52.0%	63.6%
Total		Count	281	11
		% within Place_of_residence	96.2%	3.8%
		% within I feel like I am too old to donate my organs:	100.0%	100.0%

Crosstab

			Total
Place_of_residence	Urban	Count	139
		% within Place_of_residence	100.0%
		% within I feel like I am too old to donate my organs:	47.6%
	Rural	Count	153
		% within Place_of_residence	100.0%
		% within I feel like I am too old to donate my organs:	52.4%
Total		Count	292
		% within Place_of_residence	100.0%
		% within I feel like I am too old to donate my organs:	100.0%

Chi-Square Tests

	Value	df	Asymptotic Significance (2-sided)	Exact Sig. (2-sided)	Exact Sig. (1-sided)
Pearson Chi-Square	.579[a]	1	.447		
Continuity Correction[b]	.205	1	.650		
Likelihood Ratio	.588	1	.443		
Fisher's Exact Test				.546	.328
Linear-by-Linear Association	.577	1	.448		
N of Valid Cases	292				

a. 0 cells (0.0%) have expected count less than 5. The minimum expected count is 5.24.

b. Computed only for a 2x2 table

Place_of_residence * I feel my medical co-morbidities prevent me from donating my organs:

Crosstab

			I feel my medical co-morbidities prevent me from donating my organs:	
			No, I do not have such feelings.	Yes
Place_of_residence	Urban	Count	135	4
		% within Place_of_residence	97.1%	2.9%
		% within I feel my medical co-morbidities prevent me from donating my organs:	48.6%	28.6%
	Rural	Count	143	10
		% within Place_of_residence	93.5%	6.5%
		% within I feel my medical co-morbidities prevent me from donating my organs:	51.4%	71.4%
Total		Count	278	14
		% within Place_of_residence	95.2%	4.8%
		% within I feel my medical co-morbidities prevent me from donating my organs:	100.0%	100.0%

Crosstab

			Total
Place_of_residence	Urban	Count	139
		% within Place_of_residence	100.0%
		% within I feel my medical co-morbidities prevent me from donating my organs:	47.6%
	Rural	Count	153
		% within Place_of_residence	100.0%
		% within I feel my medical co-morbidities prevent me from donating my organs:	52.4%
Total		Count	292
		% within Place_of_residence	100.0%
		% within I feel my medical co-morbidities prevent me from donating my organs:	100.0%

Chi-Square Tests

	Value	df	Asymptotic Significance (2-sided)	Exact Sig. (2-sided)	Exact Sig. (1-sided)
Pearson Chi-Square	2.135[a]	1	.144		
Continuity Correction[b]	1.409	1	.235		
Likelihood Ratio	2.215	1	.137		
Fisher's Exact Test				.176	.117
Linear-by-Linear Association	2.128	1	.145		
N of Valid Cases	292				

a. 0 cells (0.0%) have expected count less than 5. The minimum expected count is 6.66.

b. Computed only for a 2x2 table

Place_of_residence * I feel the surgery for donating organs will disfigure my body:

Crosstab

			I feel the surgery for donating organs will disfigure my body:	
			No, I do not have such feelings.	Yes
Place_of_residence	Urban	Count	133	6
		% within Place_of_residence	95.7%	4.3%
		% within I feel the surgery for donating organs will disfigure my body:	48.4%	35.3%
	Rural	Count	142	11
		% within Place_of_residence	92.8%	7.2%
		% within I feel the surgery for donating organs will disfigure my body:	51.6%	64.7%
Total		Count	275	17
		% within Place_of_residence	94.2%	5.8%
		% within I feel the surgery for donating organs will disfigure my body:	100.0%	100.0%

Crosstab

			Total
Place_of_residence	Urban	Count	139
		% within Place_of_residence	100.0%
		% within I feel the surgery for donating organs will disfigure my body:	47.6%
	Rural	Count	153
		% within Place_of_residence	100.0%
		% within I feel the surgery for donating organs will disfigure my body:	52.4%
Total		Count	292
		% within Place_of_residence	100.0%
		% within I feel the surgery for donating organs will disfigure my body:	100.0%

Chi-Square Tests

	Value	df	Asymptotic Significance (2-sided)	Exact Sig. (2-sided)	Exact Sig. (1-sided)
Pearson Chi-Square	1.096[a]	1	.295		
Continuity Correction[b]	.635	1	.426		
Likelihood Ratio	1.116	1	.291		
Fisher's Exact Test				.328	.214
Linear-by-Linear Association	1.093	1	.296		
N of Valid Cases	292				

a. 0 cells (0.0%) have expected count less than 5. The minimum expected count is 8.09.

b. Computed only for a 2x2 table

Place_of_residence * I feel my family won’t support my decision to donate my organs:

Crosstab

			I feel my family won’t support my decision to donate my organs:	
			no / NA	yes
Place_of_residence	Urban	Count	119	20
		% within Place_of_residence	85.6%	14.4%
		% within I feel my family won’t support my decision to donate my organs:	47.6%	47.6%
	Rural	Count	131	22
		% within Place_of_residence	85.6%	14.4%
		% within I feel my family won’t support my decision to donate my organs:	52.4%	52.4%
Total		Count	250	42
		% within Place_of_residence	85.6%	14.4%
		% within I feel my family won’t support my decision to donate my organs:	100.0%	100.0%

Crosstab

			Total
Place_of_residence	Urban	Count	139
		% within Place_of_residence	100.0%
		% within I feel my family won’t support my decision to donate my organs:	47.6%
	Rural	Count	153
		% within Place_of_residence	100.0%
		% within I feel my family won’t support my decision to donate my organs:	52.4%
Total		Count	292
		% within Place_of_residence	100.0%
		% within I feel my family won’t support my decision to donate my organs:	100.0%

Chi-Square Tests

	Value	df	Asymptotic Significance (2-sided)	Exact Sig. (2-sided)	Exact Sig. (1-sided)
Pearson Chi-Square	.000[a]	1	.998		
Continuity Correction[b]	.000	1	1.000		
Likelihood Ratio	.000	1	.998		
Fisher's Exact Test				1.000	.565
Linear-by-Linear Association	.000	1	.998		
N of Valid Cases	292				

a. 0 cells (0.0%) have expected count less than 5. The minimum expected count is 19.99.

b. Computed only for a 2x2 table

Place_of_residence * I have concerns that my organs will be use d for medical research rather than for patients:

Crosstab

			I have concerns that my organs will be used for medical research rather than for patients:	
			No, I do not have any such concerns.	Yes
Place_of_residence	Urban	Count	109	30
		% within Place_of_residence	78.4%	21.6%
		% within I have concerns that my organs will be used for medical research rather than for patients:	46.6%	51.7%
	Rural	Count	125	28
		% within Place_of_residence	81.7%	18.3%
		% within I have concerns that my organs will be used for medical research rather than for patients:	53.4%	48.3%
Total		Count	234	58
		% within Place_of_residence	80.1%	19.9%
		% within I have concerns that my organs will be used for medical research rather than for patients:	100.0%	100.0%

Crosstab

			Total
Place_of_residence	Urban	Count	139
		% within Place_of_residence	100.0%
		% within I have concerns that my organs will be used for medical research rather than for patients:	47.6%
	Rural	Count	153
		% within Place_of_residence	100.0%
		% within I have concerns that my organs will be used for medical research rather than for patients:	52.4%
Total		Count	292
		% within Place_of_residence	100.0%
		% within I have concerns that my organs will be used for medical research rather than for patients:	100.0%

Chi-Square Tests

	Value	df	Asymptotic Significance (2-sided)	Exact Sig. (2-sided)	Exact Sig. (1-sided)
Pearson Chi-Square	.493[a]	1	.483		
Continuity Correction[b]	.308	1	.579		
Likelihood Ratio	.492	1	.483		
Fisher's Exact Test				.557	.289
Linear-by-Linear Association	.491	1	.483		
N of Valid Cases	292				

a. 0 cells (0.0%) have expected count less than 5. The minimum expected count is 27.61.

b. Computed only for a 2x2 table

Place_of_residence * I have concerns that my organs will not go to those patients who need it most:

Crosstab

			I have concerns that my organs will not go to those patients who need it most:	
			No, I do not have any such concerns.	Yes
Place_of_residence	Urban	Count	99	40
		% within Place_of_residence	71.2%	28.8%
		% within I have concerns that my organs will not go to those patients who need it most:	45.2%	54.8%
	Rural	Count	120	33
		% within Place_of_residence	78.4%	21.6%
		% within I have concerns that my organs will not go to those patients who need it most:	54.8%	45.2%
Total		Count	219	73
		% within Place_of_residence	75.0%	25.0%
		% within I have concerns that my organs will not go to those patients who need it most:	100.0%	100.0%

Crosstab

			Total
Place_of_residence	Urban	Count	139
		% within Place_of_residence	100.0%
		% within I have concerns that my organs will not go to those patients who need it most:	47.6%
	Rural	Count	153
		% within Place_of_residence	100.0%
		% within I have concerns that my organs will not go to those patients who need it most:	52.4%
Total		Count	292
		% within Place_of_residence	100.0%
		% within I have concerns that my organs will not go to those patients who need it most:	100.0%

Chi-Square Tests

	Value	df	Asymptotic Significance (2-sided)	Exact Sig. (2-sided)	Exact Sig. (1-sided)
Pearson Chi-Square	2.018[a]	1	.155		
Continuity Correction[b]	1.652	1	.199		
Likelihood Ratio	2.018	1	.155		
Fisher's Exact Test				.177	.099
Linear-by-Linear Association	2.011	1	.156		
N of Valid Cases	292				

a. 0 cells (0.0%) have expected count less than 5. The minimum expected count is 34.75.

b. Computed only for a 2x2 table

Place_of_residence * My religious beliefs do not permit me to donate my organs

Crosstab

			My religious beliefs do not permit me to donate my organs	
			No, I have no such religious restrictions.	Yes
Place_of_residence	Urban	Count	132	7
		% within Place_of_residence	95.0%	5.0%
		% within My religious beliefs do not permit me to donate my organs	46.8%	70.0%
	Rural	Count	150	3
		% within Place_of_residence	98.0%	2.0%
		% within My religious beliefs do not permit me to donate my organs	53.2%	30.0%
Total		Count	282	10
		% within Place_of_residence	96.6%	3.4%
		% within My religious beliefs do not permit me to donate my organs	100.0%	100.0%

Crosstab

			Total
Place_of_residence	Urban	Count	139
		% within Place_of_residence	100.0%
		% within My religious beliefs do not permit me to donate my organs	47.6%
	Rural	Count	153
		% within Place_of_residence	100.0%
		% within My religious beliefs do not permit me to donate my organs	52.4%
Total		Count	292
		% within Place_of_residence	100.0%
		% within My religious beliefs do not permit me to donate my organs	100.0%

Chi-Square Tests

	Value	df	Asymptotic Significance (2-sided)	Exact Sig. (2-sided)	Exact Sig. (1-sided)
Pearson Chi-Square	2.082[a]	1	.149		
Continuity Correction[b]	1.256	1	.262		
Likelihood Ratio	2.124	1	.145		
Fisher's Exact Test				.201	.131
Linear-by-Linear Association	2.075	1	.150		
N of Valid Cases	292				

a. 1 cells (25.0%) have expected count less than 5. The minimum expected count is 4.76.

b. Computed only for a 2x2 table
