# Supplementary material for: Devising focused strategies to improve organ donor registrations: A cross-sectional study among professional drivers in coastal South India
Source: PLoS One. 2018 Dec 21;13(12):e0209686. doi: 10.1371/journal.pone.0209686 (PMC6303053; doi:10.1371/journal.pone.0209686)
Supplement: S1 Table — (DOCX) [file pone.0209686.s002.docx]

**S1 Table. Survey questions, and absolute and relative (%) number of expected responses i.e. correct, affirmative, or positive responses, on knowledge and attitude, regarding organ donation:**

| **Questions asked:** | | **Expected responses:** | **n (%) with score = 1** |
| --- | --- | --- | --- |
| **Knowledge parameters** | | | |
| 1. *Are you aware of the concept of organ donation?* | | Yes, I am aware. | 292 (97.33%) |
| 2. *When do you think one donate their organs?* | | At least one of the following: when alive; after death (cadaveric or brain dead); | 142 (47.33%)*  133 (44.33%)* |
| 3. *When do you think the following organs can be donated:* | | |  |
|  | - Kidneys | At least one of the following for each organ:  when alive; after death (cadaveric or brain dead); | 159 (53.00%)  100 (33.33%)* |
|  | - Liver |  | 147 (49.00%)  58 (19.33%)* |
|  | - Skin |  | 80 (26.67%)  52 (17.33%)* |
|  | - Lung |  | 96 (32%)  18 (6%)* |
|  | - Intestines |  | 76 (25.33%)  30 (10%)* |
|  | - Pancreas |  | 50 (16.67%)  22 (7.33%)* |
|  | - Heart | After death (cadaveric or brain dead) | 166 (55.33%) |
|  | - Cornea |  | 239 (79.67%) |
| *4. Do you think living organ donations involve any health risks for the donor?* | | Yes, living donors do pose themselves to certain health risks. | 40 (13.33%) |
| *5. In India, when alive, is it legal to donate your organs to people that are unrelated and unknown to you? i.e. are living altruistic (directed / non-directed) donations legal?* | | Yes, it is legal. | 160 (53.33%) |
| *6. In India, is it legal for the donor or their families (in case of deceased donor transplants) to accept monetary or other benefits from the recipient?* | | No, it is illegal. | 211 (70.33%) |
| **Attitude parameters: Section – I (Preferences with respect to organ donation)** | | | |
| *1. Would you extend your support to your kin if they decided to become organ donors?* | | Yes, I would. | 275 (91.67%) |
| *2. Would you give your consent for non-directed altruistic donation of a deceased relatives’ organs?* | |  | 219 (73%) |
| *3. Would you like to donate your organs?* | |  | 211 (70.33%) |
| *4. Would you be willing to donate your organs to an unrelated and unknown recipient? OR would you be comfortable donating your organs only to family and close friends.* | | I am willing to donate my organs to anyone. | 200 (66.67%) |
| *5. Does the age of the recipient influence your decision, to donate your organs or consent to the donation of a deceased relatives’ organs?* | | No, it does not; I would donate my organs irrespective of the recipients’ age or mental status. | 150 (50%) |
| *6. Does the mental status of the recipient influence your decision, to donate your organs or consent to the donation of a deceased relatives’ organs?* | |  | 124 (41.33%) |
| *7. Does the severity of the medical condition of the recipient matter influence your decision to, donate your organs or consent to the donation of a deceased relatives’ organs?* | | No, it does not; I would donate my organs irrespective of the severity of the recipients’ medical condition, so long as there exists a legitimate need for the transplant. | 109 (36.33%) |
| 8. *Does the recipients’ religion influence your decision to, donate your organs or consent to the donation of a deceased relatives’ organs?* | | No, it does not; I would donate my organs irrespective of the recipients’ religious beliefs. | 206 (68.67%) |
| **Attitude parameters: Section – II (Barriers to organ donation)** | | | |
| *1. I feel like I am too old to donate my organs:* | | No, I do not have such feelings. | 281 (93.67%) |
| *2. I feel my medical co-morbidities prevent me from donating my organs:* | |  | 278 (92.67%) |
| *3. I feel the surgery for donating organs will disfigure my body:* | |  | 275 (91.67%) |
| *4. I feel my family won’t support my decision to donate my organs:* | |  | 250 (83.33%) |
| *5. I have concerns that my organs will be used for medical research rather than for patients:* | | No, I do not have any such concerns. | 234 (78%) |
| *6. I have concerns that my organs will not go to those patients who need it most:* | |  | 219 (73%) |
| *7. My religious beliefs do not permit me to donate my organs* | | No, I have no such religious restrictions. | 282 (94%) |

*participants with a score of 2 for the concerned question.
