# Supplementary material for: Devising focused strategies to improve organ donor registrations: A cross-sectional study among professional drivers in coastal South India
Source: PLoS One. 2018 Dec 21;13(12):e0209686. doi: 10.1371/journal.pone.0209686 (PMC6303053; doi:10.1371/journal.pone.0209686)
Supplement: S2 Table — (DOCX) [file pone.0209686.s003.docx]

**S2 Table. Survey questions, and correct, affirmative, or positive responses on knowledge, attitude, and practices regarding organ donation with a detailed breakdown of scores or points**

| **Questions asked:** | **Correct, Affirmative, or Positive responses:** | **Permissible Scores or Points** | | |
| --- | --- | --- | --- | --- |
|  |  | **Min** | **Max** | |
| **Knowledge parameters** | | | | |
| 1. *Are you aware of the concept of organ donation?* | Yes, I am aware. | 0 | 1 | |
| 2. *When do you think one donate their organs?* | At least one of the following: when alive; after death (cadaveric or brain dead); | 0 | (1+1) | |
| 3. *When do you think the following organs can be donated:* | |  | | |
| - Kidneys | At least one of the following: when alive; after death (cadaveric or brain dead); | 0 | (1+1)*6 | |
| - Liver |  |  |  |  |
| - Skin |  |  |  |  |
| - Lung |  |  |  |  |
| - Intestines |  |  |  |  |
| - Pancreas |  |  |  |  |
| - Heart | After death (cadaveric or brain dead) | 0 | (1)*2 | |
| - Cornea |  |  |  |  |
| *4. Do you think living organ donations involve any health risks for the donor?* | Yes, living donors do pose themselves certain health risks. | 0 | 1 | |
| *5. In India, when alive, is it legal to donate your organs to people that are unrelated and unknown to you? i.e. are living altruistic (directed / non-directed) donations illegal in India?* | Yes, it is legal. | 0 | 1 | |
| *6. In India, is it legal for the donor or their families (in case of deceased donor transplants) to accept monetary or other benefits from the recipient?* | No, it is illegal. | 0 | 1 | |
| *Permissible range of knowledge parameter scores:* | | 0 | 20 | |
| **Attitude parameters: Section – I (Preferences with respect to organ donation)** | | | | |
| *1. Would you extend your support to your kin if they decided to become organ donors?* | Yes, I would. | 0 | | (1)*3 |
| *2. Would you give your consent for non-directed altruistic donation of a deceased relatives’ organs?* |  |  |  |  |
| *3. Would you like to donate your organs (living / deceased donor transplants)?* |  |  |  |  |
| *4. Would you be willing to donate your organs to an unrelated and unknown recipient? OR would you be comfortable donating your organs ONLY to family and close friends.* | I am willing to donate my organs to anyone. | 0 | | 1 |
| *5. Does the age of the recipient influence your decision, to donate your organs or consent to the donation of a deceased relatives’ organs?* | No, it does not; I would donate my organs irrespective of the recipients’ age or mental status. | 0 | | (1)*2 |
| *6. Does the mental status of the recipient influence your decision, to donate your organs or consent to the donation of a deceased relatives’ organs?* |  |  |  |  |
| *7. Does the severity of the medical condition of the recipient matter influence your decision to, donate your organs or consent to the donation of a deceased relatives’ organs?* | No, it does not; (I would donate my organs irrespective of the severity of the recipients’ medical condition, so long as there exists a legitimate need for the transplant.) | 0 | | 1 |
| 8. *Does the recipients’ religion influence your decision to, donate your organs or consent to the donation of a deceased relatives’ organs?* | No, it does not; (I would donate my organs irrespective of the recipients’ religious beliefs.) | 0 | | 1 |
| **Attitude parameters: Section – II (Barriers to organ donation)** | | | | |
| *1. I feel like I am too old to donate my organs:* | No, I do not have such feelings. | 0 | | (1)*4 |
| *2. I feel my medical co-morbidities prevent me from donating my organs:* |  |  |  |  |
| *3. I feel the surgery for donating organs will disfigure my body:* |  |  |  |  |
| *4. I feel my family won’t support my decision to donate my organs:* |  |  |  |  |
| *5. I have concerns that my organs will be used for medical research rather than for patients:* | No, I do not have any such concerns. | 0 | | (1)*2 |
| *6. I have concerns that my organs will not go to those patients who need it most:* |  |  |  |  |
| *7. My religious beliefs do not permit me to donate my organs* | No, I have no such religious restrictions. | 0 | | 1 |
| *Permissible range of attitude parameter scores* | | 0 | | 15 |
