# Supplementary material for: Devising focused strategies to improve organ donor registrations: A cross-sectional study among professional drivers in coastal South India
Source: PLoS One. 2018 Dec 21;13(12):e0209686. doi: 10.1371/journal.pone.0209686 (PMC6303053; doi:10.1371/journal.pone.0209686)
Supplement: S3 Table — a Unfavourable responses on knowledge parameters; b Unfavourable responses on attitude parameters; c Calculated when all 292 responses were included in the regression model. (DOCX) [file pone.0209686.s004.docx]

**S3 Table. Analysis of the outliers excluded from the logistic regression model for the predictors of willingness to sign up for an organ-donor card.**

| **Predictor variables included in the regression model** | **Outliers:** | | | | | |
| --- | --- | --- | --- | --- | --- | --- |
|  | **#1** | **#2** | **#3** | **#4** | **#5** | **#6** |
| **Predictor variables:**  **Knowledge or Attitude parameters:** | **Participants’ responses** | | | | | |
| *1. Do you think it is possible to donate one’s organs after death? i.e. are deceased donor (cadaveric or brain dead) transplants possible?* | No ^a^ | No ^a^ | Yes | Yes | Yes | Yes |
| *2. In India, is it illegal for the donor or their families (in case of deceased donor transplants) to accept monetary or other benefits from the recipient?* | No ^a^ | No ^a^ | Yes | Yes | No ^a^ | No ^a^ |
| *3. If asked to donate organs from a deceased close family member, would you agree?* | No ^b^ | No ^b^ | No ^b^ | Yes | Yes | No ^b^ |
| *4. If you were to donate your organs, what sort of donation would you prefer?* | Not Applicable – I do not wish to donate ^b^ | | | | | |
| *5. I feel my family may not support my decision to donate my organs:* | No | No | No | No | No | No |
| *6. I have concerns that my organs be used for medical research rather than for patients:* | Yes ^b^ | No | Yes ^b^ | Yes ^b^ | No | No |
| *7. I have concerns that my organs will not go to those patients who need it most:* | Yes ^b^ | No | Yes ^b^ | Yes ^b^ | No | Yes ^b^ |
| **Outcome variable:**  *Are you willing to sign up for an organ donor card?* | **Observed responses** | | | | | |
|  | Yes | Yes | Yes | Yes | Yes | Yes |
|  | **Predicted responses** | | | | | |
|  | No | No | No | No | No | No |
|  | **Studentized residuals** ^c^ | | | | | |
|  | 13.54 | 10.54 | 3.71 | 3.44 | 3.21 | 2.66 |

^a^ Unfavourable responses on knowledge parameters; ^b^ Unfavourable responses on attitude parameters; ^c^ Calculated when all 292 responses were included in the regression model;
